# Double Braking Effects of Nanomedicine on Mitochondrial Permeability Transition Pore for Treating Idiopathic Pulmonary Fibrosis

**DOI:** 10.1002/advs.202405406

**Published:** 2024-10-30

**Authors:** An Lu, Zhiyi Xu, Zhixia Zhao, Yi Yan, Linxia Jiang, Jing Geng, Hongwei Jin, Xiangyu Wang, Xiaoyan Liu, Yuanjun Zhu, Yujie Shi, Lihong Liu, Huaping Dai, Jian‐Cheng Wang

**Affiliations:** ^1^ Beijing Key Laboratory of Molecular Pharmaceutics and New Drug Delivery Systems State Key Laboratory of Natural and Biomimetic Drugs School of Pharmaceutical Sciences Peking University Beijing 100191 China; ^2^ Department of Pharmacy Clinical Trial Research Center China‐Japan Friendship Hospital Beijing 100029 China; ^3^ National Center for Respiratory Medicine State Key Laboratory of Respiratory Health and Multimorbidity National Clinical Research Center for Respiratory Diseases Institute of Respiratory Medicine Chinese Academy of Medical Sciences Peking Union Medical College Department of Pulmonary and Critical Care Medicine, Center of Respiratory Medicine China‐Japan Friendship Hospital Beijing 100029 China; ^4^ Laboratory of Innovative Formulations and Pharmaceutical Excipients Peking University Ningbo Institute of Marine Medicine Ningbo 315832 China

**Keywords:** cyclosporin A, idiopathic pulmonary fibrosis, ionizable lipid, lipid nanoparticle, siRNA delivery

## Abstract

Mitochondrial permeability transition pore (mPTP) opening is a key hallmark of injured type II alveolar epithelial cells (AECIIs) in idiopathic pulmonary fibrosis (IPF). Inhibiting mPTP opening in AECIIs is considered a potential IPF treatment. Herein, a “double braking” strategy on mPTP by cyclosporin A (CsA) derived ionizable lipid with 3D structure (3D‐lipid) binding cyclophilin D (CypD) and siRNA downregulating mitochondrial calcium uniporter (MCU) expression is proposed for treating IPF. 3D‐lipid and MCU targeting siRNA (siMCU) are co‐assembled to form stable 3D‐LNP/siMCU nanoparticles (NPs), along with helper lipids. In vitro results demonstrated that these NPs effectively inhibit mPTP opening by 3D‐lipid binding with CypD and siRNA downregulating MCU expression, thereby decreasing damage‐associated molecular patterns (DAMPs) release and suppressing epithelial‐to‐mesenchymal transition (EMT) process in bleomycin‐induced A549 cells. In vivo results revealed that 3D‐LNP/siMCU NPs effectively ameliorated collagen deposition, pro‐fibrotic factors secretion, and fibroblast activation in bleomycin‐induced pulmonary fibrosis (PF) mouse models. Moreover, compared to the commercial MC3‐based formulation, optimized Opt‐MC3/siRNA NPs with incorporating 3D‐lipid as the fifth component, showed superior therapeutic efficacy against PF due to their enhanced stability and higher gene silencing efficiency. Overall, the nanomedicine containing 3D‐lipid and siMCU will be a promising and potential approach for IPF treatment.

## Graphical abstract

Cyclosporin A derived ionizable lipid(3D‐lipid) and mitochondrial calcium uniporter (MCU) targeting siRNA (siMCU) are co‐assembled to form stable 3D‐LNP/siMCU nanoparticles, along with helper lipids. This “double braking” effects of 3D‐lipid binding with cyclophilin D (CypD) and siRNA downregulating MCU expression on mitochondrial permeability transition pore (mPTP) opening will synergistically suppress pro‐fibrotic factors secretion and fibroblast activation.

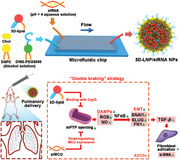

## 1 Introduction

Idiopathic pulmonary fibrosis (IPF) is a rapidly progressive and fatal alveolar disease with a median survival rate of 2–3 years.^[^ ^1^ ^]^ It is characterized by the excessive proliferation of fibroblasts, the excessive extracellular matrix deposition and the destruction of alveolar structure.^[^ ^2^ ^]^ According to World IPF Joint Association data, ≈1.22 million new cases are suffering from IPF every year.^[^ ^3^ ^]^ Environmental pollution, viral infections, and smoking are recognized as the primary potential pathogenic factors for IPF.^[^ ^4^ ^]^ Although antifibrotic drugs such as pirfenidone (PFD) and nintedanib can inhibit the progression of IPF, the unclear pathogenesis cause IPF difficult to treat or cure. Therefore, it's urgently needed to develop novel treatment strategies for IPF.

The pathogenesis of IPF is linked to repeated micro‐damage of alveolar epithelial cells (AECs), particularly type II alveolar epithelial cells (AECIIs).^[^ ^5^, ^6^ ^]^ In bleomycin (BLM)‐induced pulmonary fibrosis (PF) models, injured and dysfunctional AECIIs undergo epithelial‐to‐mesenchymal transition (EMT), leading to excessive extracellular matrix deposition and fibroblast activation.^[^ ^7^ ^]^ Growing evidences have shown that the released damage‐associated molecular patterns (DAMPs), such as ROS, from mitochondrion induces inflammation and EMT through the activation of the NF‐*κ*B/NLRP3 axis to accelerate the progression of PF.^[^ ^8^ ^]^ Mitochondrial dysfunction is a key hallmark of injured AECIIs in IPF pathogenesis, marked by the opening of the mitochondrial permeability transition pore (mPTP) and the consequent release of DAMPs.^[^ ^9^ ^]^ mPTP is a non‐specific, voltage dependent complex pore composed of multiple proteins (cyclophilin D (CypD), adenine nucleotide transferase (ANT) and voltage‐dependent ion channel (VDAC)) located on the inner membrane of mitochondria (IMM).^[^ ^10^, ^11^ ^]^ Under pathological conditions such as aging, oxidative stress, and calcium (Ca^2+^) overload, CypD translocates from the mitochondrial matrix to the IMM and then binds with ANT, thereby facilitating the formation of tunnel‐like structure comprising VDAC and ANT.^[^ ^12^ ^]^ It has been reported that cyclosporin A (CsA) can bind with the CypD in the IMM to hinder the interaction between CypD and ANT, thereby suppressing mPTP opening and alleviating mitochondrial dysfunction in the treatment of myocardial ischemia‐reperfusion injury,^[^ ^13^ ^]^ stroke,^[^ ^14^ ^]^ traumatic brain injury.^[^ ^15^ ^]^ Pulmonary effects of CsA suppressing mPTP opening on treating IPF has not been reported. Therefore, the effects of CsA binding with CypD on suppressing mPTP opening in AECIIs might be a potential strategy for treating IPF.

Furthermore, regulating the mitochondrial Ca^2+^ (mtCa^2+^) transport provides another strategy to inhibit mPTP opening. The transport process of mtCa^2+^ is controlled by the mitochondrial calcium uniporter (MCU) complex located in the IMM.^[^ ^16^ ^]^ Under normal physiological conditions, the MCU can transport Ca^2+^ from the cytoplasm into the mitochondrial matrix to stimulate Ca^2+^‐dependent tricarboxylic acid (TCA)‐cycle dehydrogenases, thereby promoting mitochondrial adenosine triphosphate (ATP) production and supporting cellular energy demands. However, the overexpressed MCU can induce an excessive accumulation of mtCa^2+^, and then trigger the mPTP opening, ultimately causing the release of DAMPs.^[^ ^17^ ^]^ It has been reported that the pathogenesis of esophageal cancer and Parkinson's disease is associated with excessive accumulation of mtCa^2+^ induced by overexpression of MCU.^[^ ^18^ ^]^ However, the role of MCU in AECIIs from IPF patients is not known. Therefore, downregulating the MCU expression level in AECIIs provides another effective strategy for treating IPF.

**Scheme 1:**
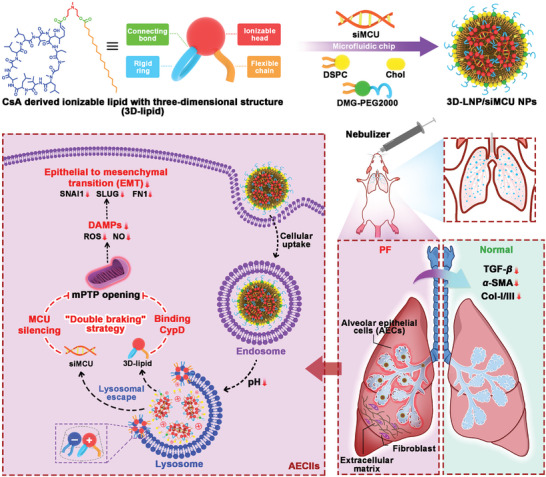
Cyclosporin A (CsA) derived ionizable lipid with 3D structure (3D‐lipid) was co‐assembled with mitochondrial calcium uniporter (MCU) targeting siRNA (siMCU) and other helper lipids using a microfluidic chip to form stable lipid nanoparticles (LNPs), termed 3D‐LNP/siMCU nanoparticles (NPs). This “double braking” effects of 3D‐lipid binding with CypD and siRNA downregulating MCU expression on mitochondrial permeability transition pore (mPTP) opening would synergistically inhibit the release of DAMPs, the EMT process of type II alveolar epithelial cells (AECIIs) and the activation of fibroblasts, thereby attenuating the progression of IPF.

Taken together the above discussion, we hypothesized that the “double braking” effects of CsA binding with CypD and siRNA downregulating MCU expression on mPTP opening would synergistically inhibit the release of DAMPs, thereby suppressing the EMT process and attenuating the progression of IPF. Interestingly, CsA derived ionizable lipids with excellent stability and lysosomal escape ability were unexpectedly discovered during their utilization in siRNA‐loaded lipid nanoparticles (LNPs) in our pre‐experiment. Here, the CsA‐derived ionizable lipid with a 3D structure (3D‐lipid) was used to package MCU targeting siRNA (siMCU) using a microfluidic chip, along with other helper lipids, to form stable 3D‐LNP/siMCU nanoparticles (NPs) for treating IPF. As shown in **Scheme** **1**, after pulmonary administration, 3D‐LNP/siMCU NPs were internalized into AECIIs and exerted an inhibition effect on mPTP opening by 3D‐lipid binding with CypD and downregulating the MCU expression, thereby suppressing the release of DAMPs and the EMT process of AECIIs. Moreover, 3D‐lipid was further incorporated into the Dlin‐MC3‐DMA (MC3)‐based commercial formulation (MC3/siRNA NPs) as the fifth component to form optimized Opt‐MC3/siRNA NPs, thereby enhancing their therapeutic potential for IPF. The in vivo therapeutic efficacy of NPs was evaluated in BLM‐induced PF mouse models. Additionally, this study provides the first evidence demonstrating the overexpression of MCU in AECIIs within the lung tissues of IPF patients.

## 2 Results and Discussion

### 2.1 The Overexpression of MCU in AECIIs of IPF

**Figure 1:**
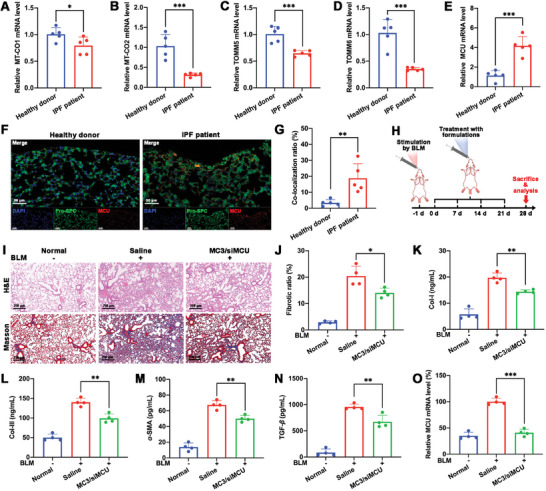
The role of MCU in AECIIs of IPF. The relative A) MT‐CO1, B) MT‐CO2, C) TOMM5, D) TOMM6, and E) MCU mRNA levels in IPF patients’ and healthy donors’ lung tissues detected using qRT‐PCR (*n* = 5); F) Representative immunofluorescence images of IPF patients’ and healthy donors’ lung tissues to analyze the expression level of MCU (red) in ACEIIs (using pro‐SPC as a marker, green). The nucleus was stained with DAPI (blue). Scale bar, 50 µm; G) Semi‐quantitation of immunofluorescence colocalization analyses in IPF patients’ and healthy donors’ lung tissues (*n* = 5); H) Schematic illustration of the treatment regimen in BLM‐induced PF mouse models; I) Representative H&E staining and Masson's trichrome staining images of lung tissues isolated from mice. Scale bar, 250 µm; J) The fibrotic ratio of lung tissues isolated from mice (*n* = 4); The expression levels of Col‐I K) and Col‐III L) in lung tissues isolated from mice detected using ELISA kits (*n* = 4); The expression levels of *α*‐SMA M) and TGF‐*β* N) in lung tissues isolated from mice detected using ELISA kits (*n* = 4); O) The relative MCU mRNA level in lung tissues isolated from mice detected using qTR‐PCR (*n* = 4). Data are represented as mean ± SD. *p < 0.05, **p < 0.01, ***p < 0.001.

Quantitative real‐time PCR (qRT‐PCR) was performed on lung tissues isolated from IPF patients to evaluate the mRNA levels of mitochondria‐related genes, including mitochondrial cytochrome c oxidase subunit 1 (MT‐CO1), mitochondrial cytochrome c oxidase subunit 2 (MT‐CO2), translocase of outer mitochondrial membrane 5 (TOMM5) and translocase of outer mitochondrial membrane 6 (TOMM6). MT‐CO1 and MT‐CO2 are genes related to ATP biosynthetic process.^[^ ^19^ ^]^ TOMM5 and TOMM6 are genes related to mitophagy.^[^ ^20^ ^]^ Compared with the lung tissues from healthy donors, lower mRNA levels of MT‐CO1 (79.4%), MT‐CO2 (31.6%), TOMM5 (64.7%), and TOMM6 (35.1%) were observed in those from IPF patients, indicating that mitochondrial dysfunction was happened in fibrotic lung cells (**Figure** **1**A–D). It has been reported that MCU overexpression could induced mtCa^2+^ overload, and thereby leading to mPTP opening and consequent mitochondrial dysfunction.^[^ ^9b^ ^]^ To verify the mitochondrial dysfunction induced by MCU overexpression, qRT‐PCR, immunohistochemistry, ELISA, and immunofluorescence colocalization analyses were used to assess MCU expression levels in lung tissues of IPF patients. As depicted in Figure 1E, the MCU mRNA level in IPF patients’ lung tissues was 3.7‐fold higher than that in healthy donors’ lung tissues. The results of immunohistochemistry and ELISA further verified the overexpression of MCU protein level in lung tissues of IPF patients (Figure S1, Supporting Information). Additionally, the co‐localization (yellow) ratio in lung tissues represents the MCU (red) expression level in AECIIs (green). As shown in Figure 1F,G, the AECIIs from dense fibrotic areas of IPF lungs exhibited 5.5‐fold higher MCU expression levels than that from healthy lung tissues. The overexpression of MCU in AECIIs within the lung tissues from IPF patients suggested that regulating the MCU expression in AECIIs might provide a potential strategy for treating IPF.

To evaluate whether downregulating the MCU expression in AECIIs could alleviate the progression of IPF, BLM‐induced PF mouse models were established (Figure 1H) to evaluate the effects of siMCU‐loaded NPs (MC3/siMCU NPs) on hydroxyproline (HYP), collagen type I (Col‐I), collagen type III (Col‐III), *α*‐smooth muscle actin (*α*‐SMA), and transforming growth factor‐*β* (TGF‐*β*). As shown in Figure 1I,J, H&E staining and Masson's trichrome staining revealed that the obvious lung tissue damage and collagen deposition in lung tissues were observed in saline group, indicating that the BLM treatment successfully induced the PF models. Notably, compared with the saline group, MC3/siMCU NPs could significantly alleviate the lung tissue damage and inhibit collagen deposition in lung tissues. HYP is a unique amino acid in collagen protein and can be used to reflect collagen metabolism and fibrosis.^[^ ^21^ ^]^ The HYP content in lung tissues was assessed by a HYP detection kit. As shown in Figure S2A (Supporting Information), the saline group showed a 1.3‐fold increase in HYP content in lung tissues compared to the normal group. MC3/siMCU NPs effectively downregulated the HYP content in lung tissues, achieving an inhibitory ratio of 34.0%. Moreover, Col‐I and Col‐III are fibrosis marker molecules.^[^ ^8^ ^]^ The expression levels of Col‐I and Col‐III in lung tissues were assessed by ELISA kits. As shown in Figure 1K,L, compared with those in the normal group, remarkably higher expression levels of Col‐I (3.4‐fold) and Col‐III (2.8‐fold) in lung tissues were observed in the saline group. The MC3/siMCU group showed lower Col‐I (73.0%) and Col‐III (70.7%) expression levels in lung tissues than the saline group. Additionally, TGF‐*β* is a key cytokine involved in lung fibroblast differentiation and pulmonary fibrosis, and can promote the activation of fibroblast into myofibroblasts. *α*‐SMA is regarded as a biomarker for myofibroblast.^[^ ^20^ ^]^ Immunohistochemistry and ELISA kits analyzed the expression levels of *α*‐SMA and TGF‐*β* in lung tissues. As shown in Figure 1M,N and Figure S2B (Supporting Information), MC3/siMCU group exhibited a lower expression levels of *α*‐SMA and TGF‐*β* in lung tissues compared to the saline group.

Considering that the lower levels of HYP, Col‐I, Col‐III, *α*‐SMA, and TGF‐*β* shown in MC3/siMCU group than the saline group, we speculate the MCU expression level is associated with the changes of these indicators. The expression level of MCU in lung tissues was accessed by qRT‐PCR, ELISA and immunofluorescence colocalization analyses. As shown in Figure 1O and Figure S2C (Supporting Information), compared with the normal group, the saline group showed a 1.9‐fold increase in MCU mRNA level and a 1.6‐fold increase in MCU protein level in lung tissues. MC3/siMCU NPs significantly downregulated the MCU mRNA level by 59.1% and the MCU protein level by 51.1%. The result of the immunofluorescence colocalization analyses further verified the significantly downregulated expression level of MCU in AECIIs by MC3/siMCU NPs (Figure S2D, Supporting Information). The overexpressed MCU in AECIIs can induce mtCa^2+^ overload, leading to mPTP opening and the consequent release of DAMPs^26^. It's reported that the excessive accumulation of ROS, one of DAMPs, can mediate the EMT process of AECIIs, thereby promoting the collagen deposition, the secretion of pro‐fibrotic factors, and the activation of fibroblasts in lung tissues.^[^ ^8^ ^]^ Taken together, the overexpression of MCU in AECIIs might play a vital role in the development of IPF, and our results demonstrated siMCU‐based delivery system could attenuate the progression of IPF.

### 2.2 Preparation and Characterization of 3D‐LNP/siRNA NPs

**Figure 2:**
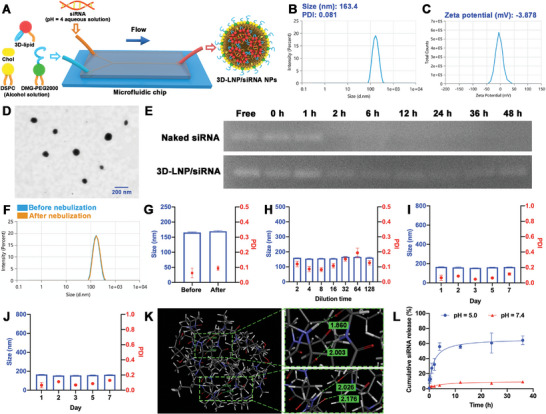
Characterization of 3D‐LNP/siRNA NP. A) Schematic illustration of the preparation process of 3D‐LNP/siRNA NPs; The particle size B) and zeta potential C) of 3D‐LNP/siRNA NPs; D) Representative STEM image of 3D‐LNP/siRNA NPs; E) The protective effect of 3D‐LNP/siRNA NPs on siRNA after incubation with FBS for different time points; F) The size distribution of 3D‐LNP/siRNA NPs before and after nebulization; G) The particle size and PDI of 3D‐LNP/siRNA NPs before and after nebulization (*n* = 3); The particle size and PDI of 3D‐LNP/siRNA NPs after a 128‐fold dilution with PBS buffer H), or 7‐day storage at 4 °C I) and 20 °C J) (*n* = 3); K) The hydrogen bonds between two 3D‐lipid molecules; L) The siRNA release profile of 3D‐LNP/siRNA NPs after mixing with anionic endosomal mimics at pH = 7.4 and 5.0 (*n* = 3). Data are represented as mean ± SD. *p < 0.05, **p < 0.01, ***p < 0.001.

To further enhance the therapeutic efficiency for IPF, a new siMCU‐based delivery system was constructed in this study. Considering the inhibitory effect of CsA on mPTP opening, a CsA derived ionizable lipid (3D‐lipid) with conjugating CsA as a side chain was synthesized and used for siRNA delivery. The 3D‐lipid and the corresponding control lipid (2D‐lipid) were synthesized as described in Figure S3 (Supporting Information). The structures of 3D‐lipid, 2D‐lipid and their intermediates were confirmed using nuclear magnetic resonance (NMR) spectrometry (Figure S4, Supporting Information) and electrospray ionization‐mass spectrometry (ESI‐MS) (Figure S5, Supporting Information). 3D‐LNP/siRNA NPs with a N/P ratio of 6/1 (mol/mol) were prepared by mixing 3D‐lipid/DSPC/Chol/DMG‐PEG (30/35/33.5/1.5, mol%) and siRNA using a microfluidic chip. As shown in **Figure** **2**A, microfluidic technology can realize the rapid mixing of two fluids using a Y‐junction microfluidic chip.^[^ ^22^ ^]^ The development of microfluidic technology improves the controllability and uniformity of LNPs.^[^ ^23^ ^]^ The corresponding control LNPs (2D‐LNP/siRNA NPs) were also prepared according to the above method by replacing 3D‐lipid with 2D‐lipid. The formulation details of 3D‐LNP/siRNA NPs and 2D‐LNP/siRNA NPs are presented in Table S1 (Supporting Information). As shown in Figure 2B,C, the 3D‐LNP/siRNA NPs with a uniform average particle size of 163.4 nm (PDI = 0.081) and a zeta potential of −3.878 mV were verified using dynamic light scattering (DLS). Meanwhile, 2D‐LNP/siRNA NPs also had a uniform average particle size of 122.7 nm (PDI = 0.1677) and a zeta potential of ‐8.049 mV (Figure S6A,B, Supporting Information). This indicated that 3D‐LNP/siRNA NPs with an excellent uniform particle size might have rigid nanoparticle structures and better protection for siRNA delivery. Scanning transmission electron microscopy (STEM) images revealed that 3D‐LNP/siRNA NPs and 2D‐LNP/siRNA NPs both showed a spherical shape (Figure 2D; Figure S6C, Supporting Information). Also, the protective effect of siRNA in LNPs was investigated using a gel retardation assay after incubation of 3D‐LNP/siRNA NPs (or 2D‐LNP/siRNA NPs) with fetal bovine serum (FBS) for different time points at 37 °C. As shown in Figure 2E and Figure S6D (Supporting Information), the band of naked siRNA disappeared in the electrophoresis images at 2 h, whereas clear bands of siRNA could be observed in the 3D‐LNP/siRNA group at 48 h and 2D‐LNP/siRNA group at 36 h. It was indicated that 3D‐LNP/siRNA NPs could have a better protection for siRNA delivery.

**Figure 3:**
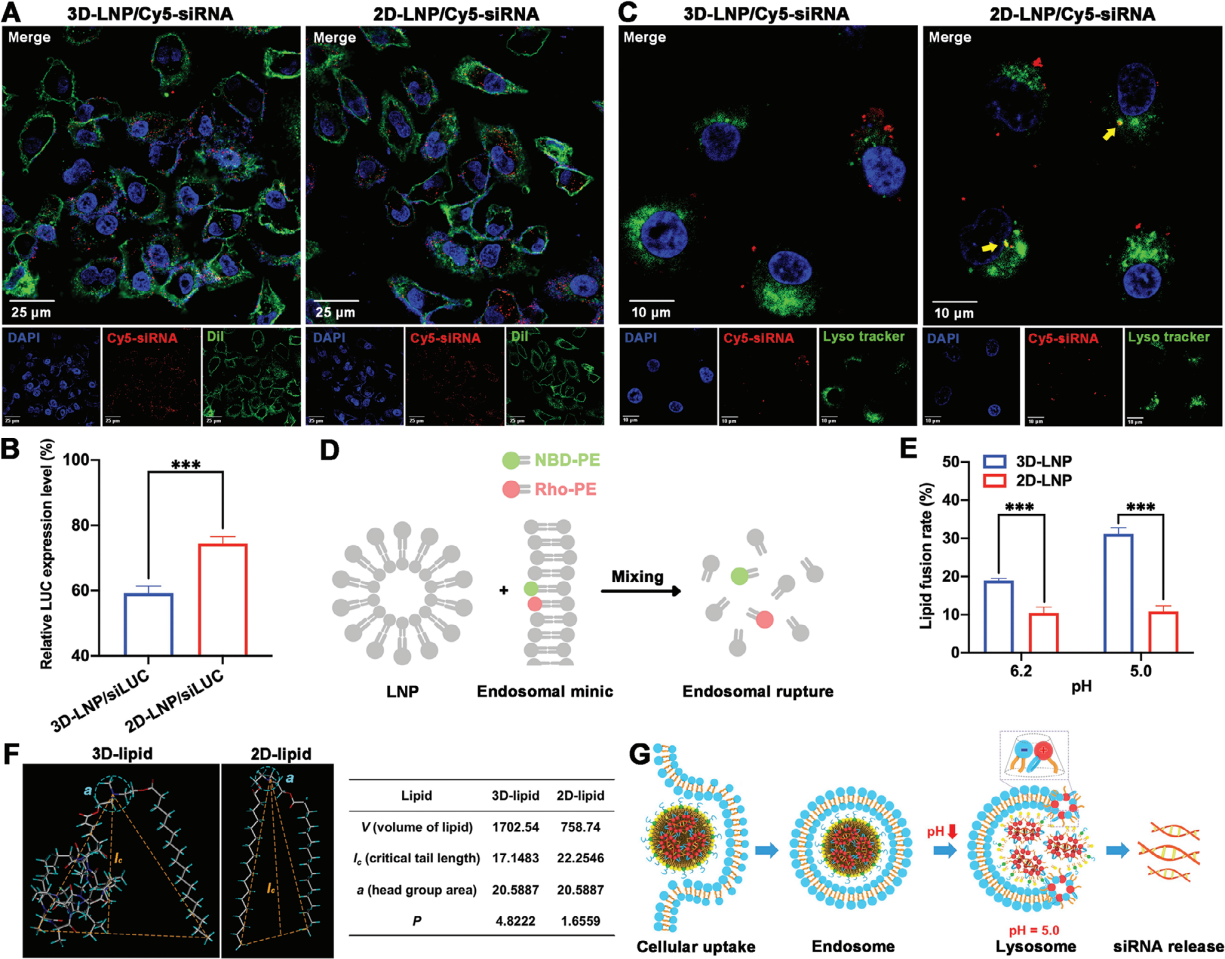
Cellular uptake, lysosomal escape and siRNA transfection of 3D‐LNP/siRNA NPs to BLM‐induced A549 cells. A) CLSM images of BLM‐induced A549 cells after incubation with Cy5‐siRNA‐loaded LNPs (red) for 6 h. The cell membrane was stained with Dil (green) and the nucleus was stained with DAPI (blue). Scale bar, 25 µm; B) The relative LUC expression level in A549‐LUC cells treated with 3D‐LNP/siLUC NPs and 2D‐LNP/siLUC NPs after nebulization (*n* = 6); C) CLSM images of co‐localization (yellow) of Cy5‐siRNA‐loaded LNPs (red) with LysoTracker Red DND‐99‐stained lysosomes (green) in BLM‐induced A549 cells after 6 h incubation. The nucleus was stained with DAPI (blue). Scale bar, 10 µm; D) Schematic illustration of the lipid fusion and membrane rupture detected by a FRET assay after mixing LNPs with anionic endosomal mimics; E) The lipid fusion rate detected by a FRET assay after mixing LNPs and anionic endosomal mimics for 10 min at pH = 6.2 and 5.0 (*n* = 3); F) Calculated parameters and *p* value of 3D‐lipid and 2D‐lipid; G) Schematic illustration of the endosomal escape mechanism of 3D‐LNP/siRNA NPs. Data represent mean ± S.D. *p < 0.05, **p < 0.01, and ***p < 0.001.

For pulmonary drug delivery, inhalation administration with a nebulizer is usually used as a potential approach to achieving the drug accumulation in lung tissue.^[^ ^24^ ^]^ However, the structures of siRNA‐loaded LNPs would be destructed by considerable shear force when passing through the nebulizer.^[^ ^25^ ^]^ To evaluate the stability of LNPs under the shear force, DLS was used to measure the size distribution of 3D‐LNP/siRNA NPs and 2D‐LNP/siRNA NPs before and after nebulization. As shown in Figure 2F,G and Figure S6E,F (Supporting Information), 3D‐LNP/siRNA NPs maintained a single‐peak size distribution after nebulization, while a dual‐peak pattern size distribution was observed in 2D‐LNP/siRNA NPs after nebulization. Compared with those in 2D‐LNP/siRNA NPs (△size = 67.0 nm, △PDI = 0.32), the changes of size and PDI in 3D‐LNP/siRNA NPs (△size = 4.0 nm, △PDI = 0.03) were significantly lower after nebulization, indicating that 3D‐LNP/siRNA NPs exhibited better stability under the shear force. In addition, the dilution stability and storage stability of NPs were also assessed using DLS. As depicted in Figure 2H and 3D‐LNP/siRNA NPs maintained a stable particle size of less than 200 nm even after a 128‐fold dilution with phosphate‐buffered saline (PBS). Interestingly, there was no change in particle size and PDI when 3D‐LNP/siRNA NPs were stored at 4 °C and 20 °C for 7 days (Figure 2I,J). Conversely, the particle size and PDI of 2D‐LNP/siRNA NPs showed obvious changes after a 128‐fold dilution with PBS (Figure S6G, Supporting Information), or during the 7‐day storage at 4 °C (Figure S6H, Supporting Information) and 20 °C (Figure S6I, Supporting Information). These findings indicate that 3D‐LNP/siRNA NPs exhibit remarkable stability, rendering them suitable for pulmonary siRNA delivery.

Furthermore, to elucidate the reason why 3D‐lipid‐based LNPs could have better stability than 2D‐lipid‐based LNPs, molecular dynamics simulation was performed with the AMBER 16 molecular simulation package. To study the elasticity of 3D‐lipid and 2D‐lipid, their dynamic structures at 0, 5, 10, 15, and 20 ns were extracted in the molecular dynamic simulation. As shown in Figure S7A (Supporting Information), the skeleton of 2D‐lipid underwent a transition from molecular stretching to molecular folding with a wider fluctuation range. Meanwhile, the skeleton of 3D‐lipid exhibited relatively lower volatility. The results of radius of gyration (*R*_g_) and root‐mean‐square deviation (RMSD) analysis further confirmed that 3D‐lipid had stronger structural rigidity than 2D‐lipid (Figure S7B–D, Supporting Information). In addition, the intermolecular interactions between two 3D‐lipid molecules were also evaluated under various binding modes, including “ring to ring,” “intersection,” “vertical” and “chain to chain” modes (Figure S7E, Supporting Information). Among them, the interaction energy of two 3D‐lipid molecules in the “ring to ring” binding mode was the lowest (‐7.99 kcal mol^−1^), indicating that 3D‐lipid molecules were most stable in this binding mode. This is attributed to forming four hydrogen bonds between two 3D‐lipid molecules (Figure 2K). Based on these results, we speculate that during the assembly of 3D‐lipid‐based LNPs, the hydrogen bonds were the driving force for 3D‐lipid molecules to adopt the “ring to ring” binding mode, and the structural rigidity and intracellular interactions of 3D‐lipid molecules enhanced the stability of siRNA‐loaded LNPs (Figure S8, Supporting Information).

The intracellular disassembly of LNPs is a crucial step for the release of siRNA and subsequent gene silencing effects. In endosomes, ionizable lipids can protonate and gain a positive charge under acidic microenvironment, the interaction between LNPs and anionic endosomal membranes induces the disassembly of LNPs and the release of siRNA.^[^ ^26^ ^]^ To investigate the pH‐responsive disassembly of LNPs, the siRNA release profile was measured after mixing LNPs and anionic endosomal mimics under different pH conditions. The anionic endosomal mimics composed of DOPS, DOPC, and DOPE was prepared by thin‐film rehydration. As shown in Figure 2L, after mixing 3D‐LNP/siRNA NPs and anionic endosomal mimics, the siRNA barely leaked from the LNPs (< 10%) at pH = 7.4, while a rapid release of siRNA occurred at pH = 5.0 (similar as pH in late endosome). Meanwhile, 2D‐LNP/siRNA NPs also exhibited a similar pH‐responsive siRNA release pattern as 3D‐LNP/siRNA NPs (Figure S9, Supporting Information).

### 2.3 siRNA Transfection of 3D‐LNP/siRNA NPs on BLM‐Induced A549 Cells

The cytotoxicity of 3D‐LNP/siMCU NPs and 2D‐LNP/siMCU NPs on human alveolar epithelial type II A549 cells was investigated using the CCK‐8 assay. As shown in Figure S10 (Supporting Information), 3D‐LNP/siMCU NPs exhibited 106.7% cell viability on A549 cells after 24 h incubation, suggesting that these LNPs had good biocompatibility in AECIIs. Meanwhile, 2D‐LNP/siMCU NPs also showed good biocompatibility in AECIIs.

Efficient cellular uptake and endosomal/lysosomal escape are the key steps for siRNA delivery in vivo. The results of flow cytometry (Figure S11, Supporting Information) and confocal laser scanning microscopy (CLSM) imaging (**Figure** **3**A) showed that 3D‐LNP/Cy5‐siRNA NPs and 2D‐LNP/Cy5‐siRNA NPs could achieve a similar fluorescence intensity on BLM‐induced A549 cells after 6 h incubation, indicating that there was no significant difference in cellular uptake between these two LNPs. Interestingly, a higher gene silencing efficiency on luciferase (LUC) was observed in A549‐LUC cells treated with 3D‐LNP/siLUC NPs (40.8%) than those treated with 2D‐LNP/siLUC NPs (25.7%) (Figure 3B).

Furthermore, to clarify the reason why 3D‐lipid‐based LNPs could have stronger gene silencing efficiency than 2D‐lipid‐based LNPs, CLSM imaging, fluorescence resonance energy transfer (FRET) assay and the calculation of packing parameter *P* value were performed. As shown in Figure 3C, BLM‐induced A549 cells treated with 3D‐LNP/Cy5‐siRNA NPs (Pearson's coefficient = 0.135) showed more red fluorescence dots (Cy5‐siRNA) separated from green fluorescence dots (lysosome stained by LysoTracker) than those treated with 2D‐LNP/Cy5‐siRNA NPs (Pearson's coefficient = 0.196) after 6 h incubation, indicating that the utilization of 3D‐lipid could facilitate the lysosomal escape of siRNA. In addition, a FRET assay was performed to evaluate the membrane‐destabilizing abilities mediated by 3D‐lipid‐based LNPs. As shown in Figure 3D,E, two FRET probes, 7‐nitrobenzo‐2‐oxa‐1,3‐diazole (NBD‐PE) and lissamine rhodamine B (Rho‐PE) were added into the anionic endosomal mimics and then mixed with LNPs at different pH conditions. Once endosomal rupture occurred, the resulting larger distance between the two FRET probes led to an NBD signal increase, which can be used to calculate the lipid fusion rate.^[^ ^27^ ^]^ Interestingly 3D‐LNP showed 1.8‐fold and 2.9‐fold higher lipid fusion rate than 2D‐LNP at pH = 6.2 and 5.0, respectively. This indicated that 3D‐lipid could induce stronger endosomal membrane destabilization than 2D‐lipid at acidic environment. It has been reported that amphiphilic lipids with an inverted conical shape could facilitate the membrane hexagonal transformation and the endosomal escape of loaded cargo.^[^ ^27^ ^]^ The packing parameter *P* is used to describe the geometry of amphiphilic lipids. Typically, an ionizable lipid with a large *P* value is favorable to promote the endosomal escape of siRNA.^[^ ^28^ ^]^ The *P* value of 3D‐lipid was calculated as: *P* = *V*/(*lc* × *a*), where *V* is the volume of the lipid, *lc* is the critical tail length and *a* is the head area of the lipid.^[^ ^29^ ^]^ As shown in Figure 3F, the *P* value of 3D‐lipid is 4.8222, which is much higher than that of 2D‐lipid (*P* = 1.6559). Based on the above results, we speculate that the enhanced lysosomal escape ability and siRNA transfection efficiency of 3D‐LNP/siRNA NPs can be attributed to the inverted conical shape of 3D‐lipid.

Taken together, after cellular uptake, 3D‐LNP/siRNA NPs are trapped in endosomes. As the pH decreases, the 3D‐lipid in lysosomes acquires a positive charge and then bonds to the anionic membrane lipids to facilitate membrane hexagonal transformation, ultimately promoting lysosomal escape of siRNA and enhancing the siRNA transfection efficiency (Figure 3G).

### 2.4 In Vitro Inhibitory Effects of 3D‐LNP/siMCU NPs on mPTP Opening

**Figure 4:**
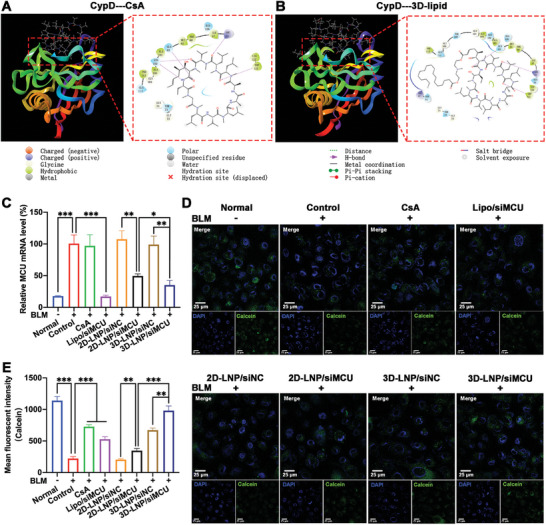
In vitro inhibitory effects of 3D‐lipid on mPTP opening. A) CsA and B) 3D‐lipid were performed the docking and molecular dynamics simulations with CypD (PDB ID: 2Z6 W); C) qRT‐PCR was used to detect the relative MCU mRNA level in BLM‐induced A549 cells treated with different formulations (*n* = 3); CLSM imaging D) and flow cytometry E) were used to detect the mPTP opening level in BLM‐induced A549 cells treated with different formulations (*n* = 3). The nucleus was stained by DAPI (blue). Scale bar, 25 µm. Data represent mean ± S.D. *p < 0.05, **p < 0.01, and ***p < 0.001.

The mPTP serves as a crucial channel for governing the material exchange and information transmission between mitochondria and cytoplasm, and is widely recognized as a key component for regulating cell damage and apoptosis.^[^ ^30^ ^]^ It has been reported that CsA could inhibit mPTP opening by binding with CypD in the IMM.^[^ ^31^ ^]^ Considering that 3D‐lipid is derived from CsA, the inhibitory effect of 3D‐lipid on mPTP opening was evaluate by the CoCl_2_‐calcein‐acetoxymethyl ester (AM) fluorescence‐quenching method. As shown in Figure S12 (Supporting Information), significantly decreased fluorescence intensity was observed in BLM‐induced A549 cells (control group) compared to A549 cells without BLM treatment (normal group), indicating that the BLM treatment enhanced the extent of mPTP opening. Compared with the control group, CsA and 3D‐lipid groups showed an equivalent increase in fluorescence intensity, whereas 2D‐lipid group did not. This indicated that the inhibitory effect of 3D‐lipid on mPTP opening was equivalent to that of CsA. To further clarify the mechanism of 3D‐lipid on inhibiting mPTP opening, the binding capability of 3D‐lipid with CypD (PDB ID: 2Z6 W) was evaluated by docking and molecular dynamics simulations. As shown in **Figure** **4**A, the double bond position was situated at the solvent‐exposed site of CsA, and its modification did not affect the binding affinity between CsA and CypD. In addition, 3D‐lipid retained most of binding sites of CsA with CypD, including Arg55, Ile57, Phe60, Met61, Gln63, Gly72, Thr73, Gly74, Ala101, Asn102, Ala103, Gly104, Gln111, Phe113, Trp121, Leu122, and His126 (Figure 4B). The interactions between 3D‐lipid and CypD involved electrostatic interaction, hydrophobic interaction, hydrogen bonding interaction and polar interaction. There was no significant difference on the capacity of CypD binding between 3D‐lipid and CsA, indicating that they would have the equivalent inhibitory effect on mPTP opening.

To evaluate the gene silencing efficiency of 3D‐LNP/siMCU NPs on MCU expression, qRT‐PCR, ELISA, and Western Blot were performed. As shown in Figure 4C, the BLM‐induced A549 cells (control group) exhibited a 5.6‐fold higher MCU mRNA level than A549 cells without the treatment of BLM (normal group). Lipo/siMCU, 2D‐LNP/siMCU, and 3D‐LNP/siMCU groups exhibited a significant gene silencing effect on MCU mRNA level in BLM‐induced A549 cells. In contrast, CsA and negative‐control siRNA (siNC)‐loaded NPs (3D‐LNP/siNC NPs and 2D‐LNP/siNC NPs) did not demonstrate any MCU mRNA level downregulation. Notably, 3D‐LNP/siMCU NPs showed significantly higher silencing efficiency on MCU mRNA level (64.9%) in BLM‐induced A549 cells compared to 2D‐LNP/siMCU NPs (51.4%). Moreover, 3D‐LNP/siMCU NPs showed dose‐dependent gene silencing effects on MCU mRNA (Figure S13A, Supporting Information). The expression level of MCU protein detected using Western Blot and ELISA was consistent with the result of qRT‐PCR (Figure S13B,C, Supporting Information). This indicated that 3D‐LNP/siMCU NPs could effectively downregulate the MCU expression level in BLM‐induced A549 cells.

To verify the activity of 3D‐LNP/siMCU NPs on inhibiting mPTP opening resulted from the synergistic effects of 3D‐lipid binding with CypD and siRNA downregulating MCU expression, the CoCl_2_‐calcein‐AM fluorescence‐quenching method was performed, and the fluorescence intensity of calcein was detected by CLSM imaging and flow cytometry. As shown in Figure 4D,E, compared with 2D‐LNP/siNC group, a higher fluorescence intensity was observed in 3D‐LNP/siNC group, indicating that the obvious mPTP opening inhibition could be exerted by 3D‐lipid binding with CypD. Moreover, 3D‐LNP/siMCU group showed higher fluorescence intensities compared to 3D‐LNP/siNC group, suggesting that downregulation of MCU expression could further enhance mPTP opening inhibition in BLM‐induced A549 cells. Therefore, we believe that the inhibition effect of 3D‐LNP/siMCU NPs on mPTP opening was associated with the synergistic mechanisms of 3D‐lipid binding with CypD and siRNA downregulating MCU expression. Based on these data, we can speculate that the synergistic effect of 3D‐LNP/siMCU NPs on mPTP opening is associated with the simultaneous inhibition of upstream (inhibiting mtCa^2+^ overload by siMCU) and downstream (binding CypD by 3D‐lipid) pathways of mPTP opening.

### 2.5 In Vitro Anti‐Pulmonary Fibrosis Mechanisms of 3D‐LNP/siMCU NPs

Growing evidences have shown that the pathogenesis of IPF is linked to the EMT process of AECIIs, which induced by mitochondrial dysfunction and the release of DAMPs, thereby leading to the secretion of TGF‐*β*.^[^ ^8^ ^]^ The enhanced TGF‐*β* secretion can facilitate the activation of fibroblasts into myofibroblasts and the excessive extracellular matrix deposition and collagen deposition.^[^ ^32^ ^]^ To elucidate the in vitro anti‐PF mechanisms of 3D‐LNP/siMCU NPs, the levels of mitochondrial dysfunction (mitochondrial membrane potential (ΔΨm) and mitochondrial ROS (mtROS)), the release of DMAPs (ROS and nitric oxide (NO)), phosphorylation and nuclear translocation of NF‐*κ*B p65, EMT (SNAI1, SLUG, and FN1 mRNA), TGF‐*β* secretion and fibroblast activation (*α*‐SMA) were measured.

**Figure 5:**
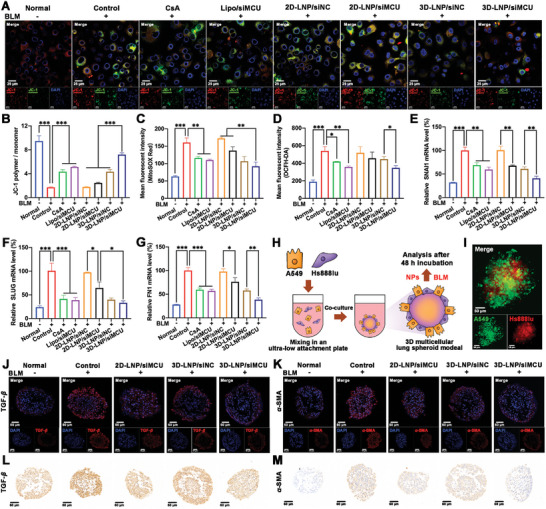
In vitro anti‐PF effects of 3D‐LNP/siMCU NPs. CLSM imaging A) and flow cytometry B) were used to detect the ΔΨm level in BLM‐induced A549 cells treated with different formulations (*n* = 3). Scale bar, 25 µm; Flow cytometry was used to detect the C) mtROS and D) ROS levels in BLM‐induced A549 cells treated with different formulations (*n* = 3); qRT‐PCR was used to detect the E) SNAI1, F) SLUG, and G) FN1 mRNA levels in BLM‐induced A549 cells treated with different formulations (*n* = 3); H) Schematic illustration of the preparation and treatment of 3D multicellular lung spheroid models; I) Fluorescence images of A549 cells (stained by Vybrant DiO, green) and Hs888lu cells (stained by Vybrant CM‐DiI, red) in 3D multicellular lung spheroid models. Scale bar, 50 µm; Representative immunofluorescence images to evaluate the expression levels of J) TGF‐*β* and K) *α*‐SMA in 3D multicellular lung spheroid models. Scale bar, 50 µm; Representative immunohistochemistry images to evaluate the expression levels of L) TGF‐*β* and M) *α*‐SMA in 3D multicellular lung spheroid models. The nucleus was stained by DAPI (blue). Data represent mean ± S.D. *p < 0.05, **p < 0.01, and ***p < 0.001.

It has been reported that inhibiting mPTP opening could effectively alleviate mitochondrial dysfunction and decrease the release of DAMPs (ROS and NO) by restoring ΔΨm and reducing mtROS.^[^ ^33^ ^]^ The JC‐1 probe was used to detect the ΔΨm. In the mitochondrial matrix, JC‐1 monomers (red) indicate a normal ΔΨm in healthy cells, while JC‐1 polymers (green) indicate a reduced ΔΨm in damaged cells.^[^ ^34^ ^]^ As shown in **Figure** **5**A,B, S14 (Supporting Information), compared with the normal group, the control group showed the conversion of red fluorescence to green fluorescence and the decrease of JC‐1 polymer/monomer ratio, indicating that BLM treatment could induce mitochondrial dysfunction with a decline in ΔΨm. Both CsA and Lipo/siMCU could increase the JC‐1 polymer/monomer ratio in BLM‐treated A549 cells, indicating that CsA binding with CypD or siRNA downregulating MCU expression could improve mitochondrial dysfunction in these cells. Notably, the 3D‐LNP/siMCU group exhibited 1.5‐fold, 2.0‐fold, and 3.0‐fold higher JC‐1 polymer/monomer ratio in BLM‐induced A549 cells than 3D‐LNP/siNC, 2D‐LNP/siMCU and 2D‐LNP/siNC groups, respectively, revealing that 3D‐LNP/siMCU NPs could exhibit the best efficiency on improving mitochondrial dysfunction by synergistic effects of 3D‐lipid and siMCU. In addition, the MitoSOX Red probe was used to detect the mtROS level. As shown in Figures 5C, S15A (Supporting Information), the control group showed a 1.9‐fold increase in mtROS level when compared to the normal group. CsA and Lipo/siMCU could effectively reduce mtROS levels in BLM‐induced A549 cells. Among the nanoformulation groups, the 3D‐LNP/siMCU group exhibited the lowest mtROS level. These results suggested that 3D‐LNP/siMCU NPs could relieve mitochondrial dysfunction by synergistic inhibition effects of 3D‐lipid and siMCU on mPTP opening.

Additionally, the ROS and NO levels in cytoplasm were used to assess the effects of 3D‐LNP/siMCU NPs on reducing DAMPs release in BLM‐induced A549 cells. DCFH‐DA and DAF‐FM DA probes were used for tracking ROS and NO, respectively. As shown in Figure 5D and Figure S15B–D (Supporting Information), compared with the normal group, a significant increase in ROS and NO levels was observed in the control group. The 3D‐LNP/siMCU group exhibited the lowest ROS and NO levels in BLM‐induced A549 cells among the nanoformulation groups. Furthermore, the 3D‐LNP/siMCU group also showed the least number of green fluorescent dots (ROS or NO) separated from mitochondrion (red) as seen in the results of CLSM imaging (Figure S16, Supporting Information). These results revealed that 3D‐LNP/siMCU NPs could effectively inhibit the release of ROS and NO from mitochondrion by inhibiting mPTP opening, thereby decreasing the accumulation of ROS and NO in the cytoplasm.

It has been reported that the excessive ROS accumulation could activate NF‐*κ*B signaling, leading to phosphorylation and nuclear translocation of NF‐*κ*B p65, ultimately resulting in the EMT process of AECIIs.^[^ ^8^ ^]^ As shown in Figure S17A (Supporting Information), compared to normal group, a 1.5‐fold increase in p‐NF‐*κ*B p65/NF‐*κ*B p65 ratio was observed in the control group. 3D‐LNP/siMCU group showed a lower p‐NF‐*κ*B p65/NF‐*κ*B p65 ratio than 3D‐LNP/siNC, 2D‐LNP/siMCU and 2D‐LNP/siMCU groups, indicating that 3D‐LNP/siMCU NPs showed the strongest inhibition effect on the phosphorylation of NF‐*κ*B p65. CLSM imaging further confirmed that 3D‐LNP/siMCU NPs inhibited the nuclear translocation of NF‐*κ*B p65 (Figure S17B, Supporting Information). These results indicated that 3D‐LNP/siMCU NPs effectively inhibit the activation of NF‐*κ*B signaling. With the inhibition in NF‐*κ*B signaling activation, the expression levels of snail family transcriptional repressor 1 (SNAI1), snail family transcriptional repressor 2 (SLUG) and fibronectin 1 (FN1) will be downregulated and the EMT process will be suppressed. SNAI1 and SLUG are key proteins in the EMT process and responsible for promoting the progression of EMT by inhibiting the expression of E‐cadherin.^[^ ^35^ ^]^ FN1 is the mesenchymal marker.^[^ ^36^ ^]^ As shown in Figure 5E–G, compared to normal group, the control group demonstrated a 2.1‐fold, 3.2‐fold and 2.6‐fold increase in the SNAI1, SLUG and FN1 mRNA levels, respectively. Both CsA and Lipo/siMCU could decrease the SLUG, FN1 and SNAI1 mRNA levels in BLM‐induced A549 cells, suggesting that 3D‐lipid binding with CypD and siRNA downregulating MCU expression could suppress the EMT process of AECIIs. Notably, 3D‐LNP/siMCU NPs exhibited the highest inhibitory rates on SNAI1 (59.1%), SLUG (66.8%), and FN1 (61.5%) mRNA levels among the nanoformulations. Overall, 3D‐LNP/siMCU NPs exerted a synergistically inhibitory effect on the EMT process of AECIIs by inhibiting NF‐*κ*B signaling activation.

The above results in 2D‐cultured cells have demonstrated that 3D‐LNP/siMCU NPs could relieve mitochondrial dysfunction and decrease the release of DAMPs, thereby inhibiting NF‐*κ*B signaling activation and the consequent EMT process of AECIIs. To further evaluate the in vitro anti‐PF effect of 3D‐LNP/siMCU NPs, 3D multicellular lung spheroid models (Figure 5H) containing A549 cells and Hs888lu cells (a human normal lung fibroblast) were established according to the method reported in literature.^[^ ^37^ ^]^ The successful establishment of 3D multicellular lung spheroid models with a diameter of 200 µm was confirmed by optical (Figure S18A, Supporting Information) and fluorescence (Figure 5I) images. Immunofluorescence and immunohistochemistry imaging were used to detect the expression levels of TGF‐*β* and *α*‐SMA in BLM‐induced 3D multicellular lung spheroid models treated with different formulations. As shown in Figure 5J,K and Figure S18B,C (Supporting Information), the control group showed higher expression levels of TGF‐*β* and *α*‐SMA than the normal group, indicating the successful inducement of fibrotic lung spheroid models. Compared with 2D‐LNP/siMCU and 3D‐LNP/siNC groups, 3D‐LNP/siMCU group showed lower expression levels of TGF‐*β* and *α*‐SMA, indicating that 3D‐LNP/siMCU NPs exerted more potent inhibition effects on the secretion of pro‐fibrotic cytokines and the activation of fibroblasts. The results of immunohistochemistry imaging further confirmed the inhibitory effects of 3D‐LNP/siMCU NPs on TGF‐*β* and *α*‐SMA expression levels (Figure 5L,M).

Overall, in vitro results demonstrated that 3D‐LNP/siMCU NPs exerted significant anti‐PF efficiency by the inhibition on mPTP opening and the release of DAMPs by 3D‐lipid binding with CypD and siRNA downregulating MCU expression. The decreased ROS level by 3D‐LNP/siMCU NPs inhibited the NF‐*κ*B pathway activation and EMT process of AECIIs, thereby suppressing the secretion of pro‐fibrotic factors and the activation of fibroblasts.

### 2.6 In Vivo Therapeutic Effects of 3D‐LNP/siMCU NPs on BLM‐Induced Pulmonary Fibrosis Mouse Models

To evaluate the retention time of LNPs in the lung tissue after pulmonary administration, an in vivo imaging system was used to detect the fluorescence biodistribution of Cy5‐siRNA‐loaded LNPs. As shown in Figure S19 (Supporting Information), robust fluorescence intensities were observed in lung tissues at 0.5 h time point after pulmonary administration of 3D‐LNP/Cy5‐siRNA NPs using a nebulizer. Notably, the obviously visible fluorescence intensity was observed for up to 7 days in lung tissues of mice treated with 3D‐LNP/Cy5‐siRNA NPs. These results indicated that3D‐LNP/Cy5‐siRNA NPs could achieve effective lung deposition within 7 days, suggesting 7‐day interval administration approach may be suitable for in vivo experiments.

**Figure 6:**
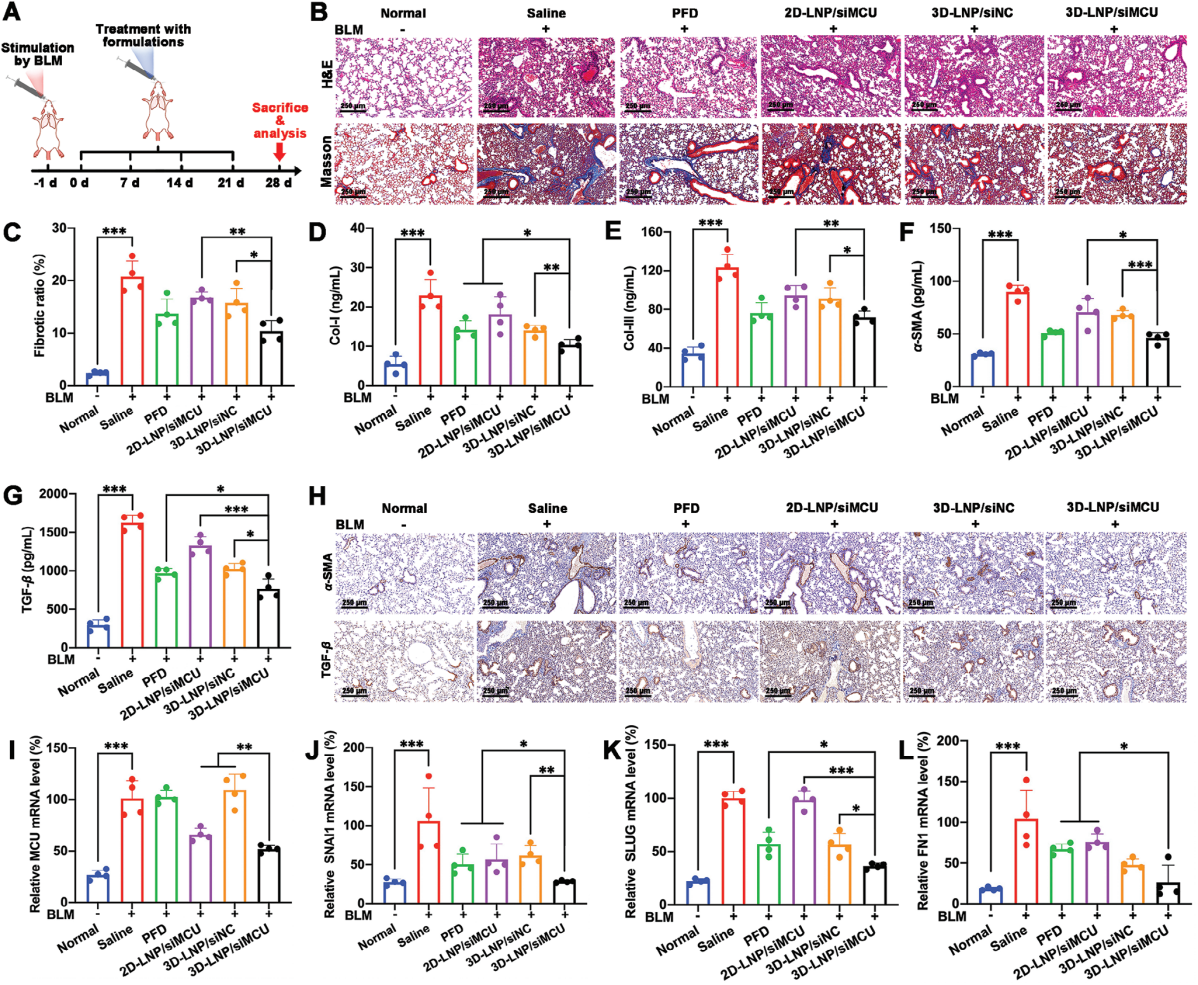
The in vivo therapeutic effect of 3D‐LNP/siMCU NPs on BLM‐induced PF mouse models. A) Schematic illustration of the treatment regimen in BLM‐induced PF mouse models; B) Representative H&E staining and Masson's trichrome staining images of lung tissues isolated from mice. Scale bar, 250 µm; C) The fibrotic ratio of lung tissues isolated from mice (*n* = 4); The expression levels of D) Col‐I, E) Col‐III, F) *α*‐SMA, and G) TGF‐*β* in lung tissues isolated from mice detected using ELISA kits (*n* = 4); (H) Representative immunohistochemistry images to evaluate the expression levels of *α*‐SMA and TGF‐*β* in lung tissues isolated from mice. Scale bar, 250 µm; The I) MCU, J) SNAI1, K) SLUG, and L) FN1 mRNA levels in lung tissues isolated from mice detected using qRT‐PCR (*n* = 4). Data represent mean ± S.D. *P < 0.05, **P < 0.01, and ***P < 0.001.

Considering that 3D‐LNP/siMCU NPs exerted excellent in vitro anti‐PF effects and lung deposition, the in vivo therapeutic effects of 3D‐LNP/siMCU NPs on fibrotic ratio, Col‐I, Col‐III, HYP, TGF‐*β*, and *α*‐SMA were further investigated on established BLM‐induced PF mouse models (**Figure** **6**A). PFD, an anti‐PF drug approved by US Food and Drug Administration (FDA),^[^ ^38^ ^]^ was used as a positive control in this study. As shown in Figure 6B,C, the results of H&E and Masson's trichrome staining revealed that 3D‐LNP/siMCU NPs could significantly alleviate the lung tissue damage and inhibit collagen deposition in lung tissues. In addition, the expression levels of Col‐I and Col‐III in lung tissues were assessed using ELISA kits. As shown in Figure 6D,E, the 3D‐LNP/siMCU group exhibited lower Col‐I and Col‐III content than 3D‐LNP/siNC and 2D‐LNP/siMCU groups. Interestingly, 3D‐LNP/siMCU group exhibited a stronger inhibition effect on the expression level of Col‐I (inhibitory rate: 54.9%) in lung tissues compared to PFD group (inhibitory rate: 38.2%), while similar effects on Col‐III (inhibitory rate: 42.0%) was observed as those in PFD group (inhibitory rate: 38.2%). In addition, a HYP detection kit was used to measure the HYP level in lung tissues. As shown in Figure S20A (Supporting Information), 3D‐LNP/siMCU group (inhibitory rate: 52.1%) exhibited a lower HYP level in the lung tissues than the 2D‐LNP/siMCU (inhibitory rate: 24.9%), 3D‐LNP/siNC (inhibitory rate: 38.0%) and PFD (inhibitory rate: 40.1%) groups. Moreover, immunohistochemistry and ELISA kits were used to analyze the expression levels of *α*‐SMA and TGF‐*β* in lung tissues. As shown in Figure 6F–H, the 3D‐LNP/siMCU NPs effectively downregulated the expression levels of *α*‐SMA and TGF‐*β* in lung tissues. Notably, the 3D‐LNP/siMCU group exhibited a lower expression level of TGF‐*β* (inhibitory rate: 53.0%) in lung tissues compared to PFD group (inhibitory rate: 40.6%), while similar effects on *α*‐SMA (inhibitory rate: 48.5%) was observed as those in PFD group (inhibitory rate: 43.2%). These results demonstrated that 3D‐LNP/siMCU NPs exhibited significant suppression on collagen deposition, the secretion of pro‐fibrotic factors, and fibroblast activation in BLM‐induced PF mouse models by synergistic effects of 3D‐lipid and siMCU.

To further investigate the in vivo anti‐PF mechanisms of 3D‐LNP/siMCU NPs, the levels of MCU mRNA and protein, malonaldehyde (MDA), hydrogen peroxide (H_2_O_2_), NO, EMT (mRNA levels of SNAI1, SLUG, and FN1) in lung tissues were detected. As shown in Figure 6I and Figure S20B (Supporting Information), 3D‐LNP/siMCU NPs (mRNA: 48.0%, protein: 36.1%) showed a stronger downregulation effect on the mRNA and protein levels of MCU in lung tissues than 2D‐LNP/siMCU NPs (mRNA: 34.0%, protein: 20.5%). In contrast, neither PFD nor 3D‐LNP/siNC NPs exhibited any significant downregulation effect on the mRNA and protein levels of MCU in lung tissues. Furthermore, the results of the immunofluorescence colocalization analyses confirmed the significantly decreased expression level of MCU in AECIIs by 3D‐LNP/siMCU NPs (Figure S20C, Supporting Information). MDA, a product of lipid oxidation by ROS, can be used as an indicator of ROS production.^[^ ^39^ ^]^ H_2_O_2_ and NO are typical substances of ROS.^[^ ^40^ ^]^ The MDA, H_2_O_2,_ and NO levels in lung tissues were measured by an MDA detection kit, a H_2_O_2_ detection kit, and Griess assay, respectively. As shown in Figure S21 (Supporting Information), compared to 3D‐LNP/siNC and 2D‐LNP/siMCU groups, lower MDA, H_2_O_2,_ and NO levels in lung tissues were observed in 3D‐LNP/siMCU group. Notably, 3D‐LNP/siMCU NPs showed a stronger inhibitory effect on MDA, H_2_O_2,_ and NO levels in lung tissues than PFD. To evaluate the effect of 3D‐LNP/siMCU NPs on the EMT process of AECIIs, qRT‐PCR was performed to measure the SNAI1, SLUG, and FN1 mRNA levels in lung tissues. As shown in Figure 6J–L, the 3D‐LNP/siMCU group exhibited lower SNAI1, SLUG, and FN1 mRNA levels in lung tissues than 3D‐LNP/siNC and 2D‐LNP/siMCU groups. Interestingly, 3D‐LNP/siMCU NPs showed a stronger inhibitory effect on the SNAI1, SLUG, and FN1 mRNA levels in lung tissues than PFD. Based on these results, it was demonstrated that the remarkable inhibition effects on the release of DAMPs and the EMT of AECIIs were attributed by 3D‐LNP/siMCU NPs, which probably associated with mPTP opening inhibition induced by the synergistic effects of 3D‐lipid binding with CypD and siRNA downregulating MCU expression.

Compared with the PFD group, 3D‐LNP/siMCU group showed lower Col‐I protein, TGF‐*β* protein, HYP, MDA, SLUG mRNA, SNAI1 mRNA, and FN1 mRNA levels, indicating that 3D‐LNP/siMCU NPs showed better therapeutic efficiency than PFD due to their synergistic effects. It has been reported that PFD exerted anti‐PF efficiency through regulating TGF‐*β*1/Smad2/3 signaling pathway.^[^ ^38^ ^]^ Although an effective therapeutic efficiency on BLM‐induced PF mouse models was confirmed in PFD group, the downstream TGF‐*β* was significantly inhibited by PFD. Different to PFD, the 3D‐LNP/siMCU NPs also exhibited significantly higher therapeutic efficiency on BLM‐induced PF mouse models through the upstream mPTP opening inhibition induced by 3D‐lipid binding with CypD and siRNA downregulating MCU expression. This indicated that the “double braking” strategy of 3D‐LNP/siMCU NPs might be more advantageous for treating IPF.

To evaluate the safety of 3D‐LNP/siMCU NPs in vivo, the H&E staining of major organs was performed at the end of treatment. As shown in Figure S22A (Supporting Information), no abnormality in major organs (liver, brain, kidney, heart, and spleen) was observed in 3D‐LNP/siMCU group. Moreover, no abnormality in the hematological indices including red blood cell (RBC), hemoglobin (HGB) and platelet (PLT) was shown in 3D‐LNP/siMCU group at the end of treatment via hematological assessments (Figure S22B–D, Supporting Information). These results indicated that pulmonary administration of 3D‐LNP/siMCU NPs had good biocompatibility for in vivo IPF therapy.

### 2.7 In Vivo Therapeutic Effects of Opt‐MC3/siMCU NPs on BLM‐Induced Pulmonary Fibrosis Mouse Models

The MC3‐based siRNA delivery system Patisiran (Onpattro), developed by Alnylam and approved by FDA in 2018, is the first commercialized siRNA therapeutic drug.^[^ ^41^ ^]^ Although the commercial MC3 ionizable lipid‐based formulation (MC3/siMCU NPs) have shown therapeutic efficacy in BLM‐induced PF mouse models (Figure 1H–O), their clinical application in treating IPF might be limited due to instability of LNPs after nebulization. Considering that 3D‐lipid could enhance the stability of LNPs, facilitate the lysosomal escape of siRNA and exert an inhibitory effect on mPTP opening, we speculate that the optimized Opt‐MC3/siRNA NPs with incorporating 3D‐lipid into the nanostructure of MC3/siRNA NPs as the fifth component would show better stability and superior therapeutic efficacy against IPF than MC3/siMCU NPs.

Opt‐3D‐LNP/siRNA NPs and MC3/siRNA NPs were prepared through microfluidic technology according to the formulation details presented in Table S2 (Supporting Information). As shown in Figure S23A,B (Supporting Information), the Opt‐MC3/siRNA NPs with a uniform average particle size of 136.2 nm (PDI = 0.0739) and a zeta potential of −6.452 mV were verified using DLS. Meanwhile, MC3/siRNA NPs also had a uniform particle size of 129.1 nm (PDI = 0.2034) and a zeta potential of −5.927 mV (Figure S23C,D, Supporting Information). Interestingly, the incorporation of 3D‐lipid facilitated the attainment of an optimized formulation with remarkably low PDI (< 0.1), indicating that Opt‐MC3/siRNA NPs might have rigid nanoparticle structures and better protection for siRNA delivery than MC3/siRNA NPs. STEM images revealed that Opt‐MC3/siRNA NPs and MC3/siRNA NPs both showed a spherical shape (Figure S23E,F, Supporting Information). To evaluate the stability of nanoparticle under the shear force, the size distribution of Opt‐MC3/siRNA NPs and MC3/siRNA NPs before and after nebulization were measured using DLS. As shown in Figure S24A–D (Supporting Information), Opt‐MC3/siRNA NPs still maintained a single‐peak size distribution after nebulization, while a dual‐peak pattern size distribution was observed in MC3/siRNA NPs after nebulization. The changes of size and PDI in Opt‐MC3/siRNA NPs (△size = 12.7 nm, △PDI = 0.01) were significantly lower after nebulization when compared with those in MC3/siRNA NPs (△size = 96.37 nm, △PDI = 0.14), indicating that the incorporation of 3D‐lipid enhanced the stability of LNPs under the shear force. Moreover, there was no significant change in particle size and PDI when Opt‐MC3/siRNA NPs were stored at 4 °C and 20 °C for 7 days. Conversely, the particle size and PDI of MC3/siRNA NPs showed obvious changes after 7‐day storage at 4 °C and 20 °C (Figure S24E–H, Supporting Information). These findings indicated that Opt‐MC3/siRNA NPs have better stability than MC3/siRNA NPs, which might be attributed to the enhanced structural rigidity and intramolecular interactions induced by 3D‐lipid molecules.

The cytotoxicity of Opt‐MC3/siMCU NPs on human alveolar epithelial type II A549 cells was investigated using the CCK‐8 assay. As shown in Figure S25 (Supporting Information), Opt‐MC3/siMCU NPs exhibited 94.5% cell viability on A549 cells after 24 h incubation, suggesting that Opt‐MC3/siMCU NPs had good biocompatibility in AECIIs. The difference of gene silencing efficiency between Opt‐MC3/siRNA NPs and MC3/siRNA NPs were evaluated using a LUC assay. As shown in Figure S26 (Supporting Information), a higher downregulation efficiency on LUC protein was observed in A549‐LUC cells treated with Opt‐MC3/siLUC NPs (50.9%) than those treated with MC3/siLUC NPs (39.7%), which might be attributed to the enhanced stability and improved endosomal escape efficiency induced by 3D‐lipid.

**Figure 7:**
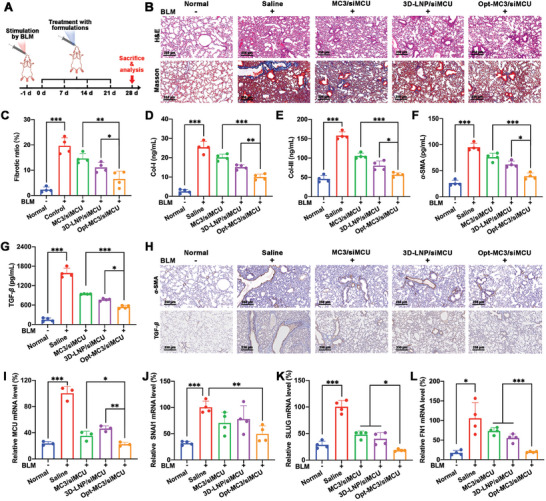
In vivo therapeutic effects of Opt‐MC3/siMCU NPs on BLM‐induced PF mouse models. A) Schematic illustration of the treatment regimen in BLM‐induced PF mouse models; B) Representative H&E staining and Masson's trichrome staining images of lung tissues isolated from mice. Scale bar, 250 µm; C) The fibrotic ratio of lung tissues isolated from mice (*n* = 4); The expression levels of D) Col‐I, E) Col‐III, F) *α*‐SMA, and G) TGF‐*β* in lung tissues isolated from mice detected using ELISA kits (*n* = 4); H) Representative immunohistochemistry images to evaluate the expression levels of *α*‐SMA and TGF‐*β* in lung tissue isolated from mice. Scale bar, 250 µm; I) The MCU mRNA level in lung tissues isolated from mice detected by qRT‐PCR (*n* = 3); The J) SNAI1, K) SLUG, and L) FN1 mRNA levels in lung tissues isolated from mice detected using qRT‐PCR (*n* = 4). Data represent mean ± S.D. *p < 0.05, **p < 0.01, and ***p < 0.001.

To evaluate whether the Opt‐MC3/siMCU NPs could exhibit better anti‐PF efficiency than MC3/siMCU NPs on BLM‐induced PF mouse models (**Figure** **7**A), the effects of Opt‐MC3/siMCU NPs and MC3/siMCU NPs on fibrotic ratio, Col‐I, Col‐III, HYP, TGF‐*β*, and *α*‐SMA were compared. The results of H&E staining and Masson's trichrome staining (Figure 7B) revealed that Opt‐MC3/siMCU NPs alleviated the lung tissue damage and inhibit the collagen deposition. Compared with the MC3/siMCU group, Opt‐MC3/siMCU group exhibited lower fibrotic ratio (Figure 7C), Col‐I and Col‐III expression levels (Figure 7D,E), and HYP level (Figure S27A, Supporting Information) in lung tissues. In addition, immunohistochemistry and ELISA kits were used to measure the expression levels of key proteins (*α*‐SMA and TGF‐*β*) related to PF. As shown in Figure 7F–H, compared with the MC3/siMCU NPs and 3D‐LNP/siMCU groups, the Opt‐MC3/siMCU group showed lower expression levels of *α*‐SMA and TGF‐*β* in lung tissues. Based on these results, Opt‐MC3/siMCU NPs showed a superior therapeutic effect on BLM‐induced PF mouse models than MC3/siMCU NPs, indicating that the incorporation of 3D‐lipid could enhance the therapeutic efficiency of MC3‐based commercial formulation for IPF.

To further investigate the anti‐PF mechanisms of Opt‐MC3/siMCU NPs, the levels of MCU, MDA, H_2_O_2_, NO, EMT (the mRNA levels of SNAI1, SLUG, and FN1) in lung tissues were detected. As shown in Figure 7I, Opt‐MC3/siMCU NPs showed a more pronounced silencing effect on MCU mRNA level (77.5%) in lung tissues compared to MC3/siMCU NPs (64.7%) and 3D‐LNP/siMCU NPs (53.6%). The MCU protein level in lung tissues detected using an ELISA kit was consistent with the result of qRT‐PCR (Figure S27B, Supporting Information). Immunofluorescence colocalization analyses further confirmed the significant downregulation of MCU expression in AECIIs by Opt‐MC3/siMCU NPs (Figure S27C, Supporting Information). As shown in Figure S28 (Supporting Information), Opt‐MC3/siMCU NPs exerted stronger inhibitory effects on MDA, H_2_O_2,_ and NO levels in lung tissues than MC3/siMCU NPs. Notably, among the nanoformulation groups, the Opt‐MC3/siMCU group exhibited the lowest SNAI1, SLUG, and FN1 mRNA levels in lung tissues (Figure 7J–L). Moreover, the result of H&E staining and blood routine test showed that no abnormality in major organs and hematological indices (RBC, HGB, and PLT) was observed in the Opt‐MC3/siMCU group (Figure S29, Supporting Information), indicating that pulmonary administration Opt‐MC3/siMCU NPs showed good biocompatibility for in vivo IPF therapy. The above results demonstrated that Opt‐MC3/siMCU NPs exhibited stronger inhibitory effects on the release of DAMPs and the EMT process of AECIIs than MC3/siMCU NPs, thereby exerting a superior therapeutic effect on BLM‐induced PF mouse models.

## 3 Conclusion

This study first demonstrated the overexpression of MCU in AECIIs in lung tissues of IPF patients. A “double braking” strategy with 3D‐lipid binding with CypD and siRNA downregulating MCU expression was proposed to inhibit mPTP opening in AECIIs, and the concept‐based 3D‐LNP/siMCU NPs was constructed for treating IPF. The in vitro and in vivo results revealed that 3D‐LNP/siMCU NPs could significantly inhibit the release of DAMPs and subsequent EMT process of AECIIs by the synergistic effects of 3D‐lipid binding with CypD and siRNA downregulating MCU expression on mPTP opening inhibition, ultimately suppressing the collagen deposition, the secretion of pro‐fibrotic factors and fibroblast activation in lung tissues. Next, the Opt‐MC3/siMCU NPs with incorporation of 3D‐lipid as the fifth component into the MC3/siMCU NPs further enhanced pulmonary siRNA delivery efficiency and anti‐PF therapeutic activity. Therefore, the “double braking” strategy of nanomedicine based on 3D‐lipid binding with CypD and siRNA downregulating MCU expression would be a promising and potential approach for treating IPF.

## 4 Experimental Section

### Reagents

Dichloromethane (DCM), ethanol, and methanol (MeOH) were obtained from Beijing Tong Guang Fine Chemicals Company (Beijing, China). Octadecanoic acid and 3‐(ethyliminomethylideneamino)‐N,N‐dimethylpropan‐1‐amine, hydrochloride (EDCI) were obtained from Bide Pharmatech Ltd. (Shanghai, China); Cyclosporin A (CsA) was obtained from Shanghai Yuanye Bio‐Technology Co., Ltd (Shanghai, China); 4‐dimethylaminopyridine (DMAP) was obtained from J&K Scientific (Beijing, China); N,N‐Diisopropylethylamine (DIPEA) was obtained from Anhui Zesheng Technology Co., Ltd (Anhui, China); Dlin‐MC3‐DMA, DSPC, cholesterol, and DMG‐PEG2000 were obtained from AVR (Shanghai) Pharmaceutical Technology Co., Ltd (Shanghai, China). JC‐1 and DCFH‐DA probes were obtained from Beijing Pulilai Gene Technology Co., Ltd (Beijing, China). DAF‐FM DA probe was obtained from Shanghai Biyuntian Biotechnology Co., Ltd (Shanghai, China). siNC, siMCU, and the fluorescent (Cy5)‐labeled siRNA (the scrambled sequence is same with the siNC) were supplied by RiboBio Co. Ltd. (Guangzhou, China); PBS was obtained from Pricella Life Science&Technology Co., Ltd (Wuhan, China). The PCR primers were synthesized by synbio technologies (Suzhou, China). Enzyme‐linked immunosorbent assay (ELISA) kits to detect MCU, Col‐I *α*‐SMA, TGF‐*β*, were purchased from Dongge Boye Biotechnology Co., Ltd (Beijing, China). An ELISA kit to detect Col‐III was purchased from Beijing Mecen Unicreate Bio‐Tech Co., Ltd. The Firefly Glo Luciferase Reporter Gene Assay Kit was purchased from Yeasen Biotechnology (Shanghai) Co., Ltd (Shanghai, China). The HYP assay kit was purchased from Nanjing Jiancheng Bioengineering Institute (Nanjing, China). The nebulizer (YAN 30 012) was obtained from Shanghai Yuyan Instruments Co., Ltd (Shanghai, China).

### Patient Samples/Ethics

Human lung tissues were obtained from China‐Japan Friendship Hospital. Samples were surgical remnants isolated from donors (*n* = 5) and IPF patients who underwent pulmonary transplantation (*n* = 5). Informed consent was obtained from all patients in this study, and the study was officially approved by the Ethics Committee of China‐Japan Friendship Hospital (Licence NO. 2017–25).

### Cell Line and Animals

A549 cells were originally purchased from Institute of Basic Medical Science, Chinese Academy of Medical Sciences (Beijing, China), and cultured in DMEM medium (Macgene, Beijing, China) supplemented with 10% FBS (PAN, Germany), 100 U mL^−1^ penicillin and 0.1 mg mL^−1^ streptomycin (Macgene, Beijing, China). Hs888lu cells were purchased from Cloud Clone (Beijing) Biotechnology Co., Ltd, and culture in DMEM medium (Macgene, Beijing, China) supplemented with 15% FBS (PAN, Germany), 100 U mL^−1^ penicillin and 0.1 mg mL^−1^ streptomycin (Macgene, Beijing, China). A549‐LUC cells were purchased from iCell Bioscience Inc (Shanghai, China), and cultured in F12K medium (iCell, Shanghai, China) supplemented with 10% FBS (PAN, Germany), 100 U mL^−1^ penicillin and 0.1 mg mL^−1^ streptomycin (Macgene, Beijing, China). Cells were cultured in a humidified incubator with 5% CO_2_ at 37 °C. Cells for all experiments were in the logarithmic phase of growth.

The C57BL/6J mice (male, 6–8 weeks) were purchased from Beijing Vital River Laboratory Animal Technology Company Limited (Beijing, China) and kept in the specific pathogen‐free (SPF) environment. All animal care and experiments were approved by Institutional Authority for Laboratory Animal Care of Peking University (Licence NO. LA2022538).

### The Synthesis of 2D‐Lipid and 3D‐Lipid—Synthesis of A_0_C_18_ and 2D‐Lipid

A mixture of 2.840 g (10.0 mmol) stearic acid, 1.190 g (10.0 mmol) N‐Methyldiethanolamine, 1.220 g (10.0 mmol) DMAP, and 2.0 mL (17.2 mmol) DIPEA were dissolved into 15.0 mL DCM, and stirred for 20 min at room temperature. And then 1.910 g (10.0 mmol) EDCI dissolved in 10.0 mL DCM was slowly added to the reaction solution. The mixture solution was stirred overnight at room temperature. Then, DCM was removed under reduced pressure and the crude product was purified by column chromatography (DCM/MeOH = 300/1 → 50/1); A white solid powder of 2D‐lipid (15.5% yield) weighting 1.012 g was first eluted and obtained. ^1^H NMR (400 MHz, CDCl_3_‐*d_6_*): *δ* (ppm) 4.326‐4.165 (m, 2 H), 3.623 (m, 2 H), 2.785‐2.647 (m, 4 H), 2.384‐2.302 (m, 7 H), 1.630 (t, *J* = 6.4 Hz, 4 H), 1.345 (s, 56 H), 0.956 (m, 6 H); ESI‐MS (m/z): 652.6378 [M+H]^+^. A white powder of A_0_C_18_ (29.4% yield) weighting 1.132 g was subsequently eluted and obtained. ^1^H NMR (400 MHz, CDCl_3_‐*d_6_*): *δ* (ppm) 4.328‐4.174 (m, 2 H), 3.681 (t, *J* = 4.8 Hz, 2 H), 2.860 (t, *J* = 5.6 Hz, 2 H), 2.738 (m, 2 H), 2.462‐2.305 (m, 5 H), 1.634 (t, *J* = 6.4 H, 2 H), 1.301 (s, 28 H), 0.934 (m, 3 H); ESI‐MS (m/z): 386.3677 [M+H]^+^.

### The Synthesis of 2D‐Lipid and 3D‐Lipid—Synthesis of ACR‐A_0_C_18_

A mixture of 0.501 g A_0_C_18_ (1.3 mmol) and 0.555 mL TEA (4.0 mmol) were dissolved into 5.0 mL DCM, and stirred at room temperature. And then, 0.325 mL acryloyl chloride (4.0 mmol) were slowly added into the reaction solution. The mixture solution was stirred at room temperature for 1 h. Then, DCM was removed under reduced pressure and the crude product was purified by column chromatography (DCM/MeOH = 300/1 → 50/1) to obtain 0.318 g ACR‐A_0_C_18_ (72.2% yield) as a white solid powder. ^1^H NMR (400 MHz, CDCl_3_‐*d_6_*): *δ* (ppm) 6.433 (d, *J* = 17.2 Hz, 1 H), 6.170 (m, 1 H), 5.853 (d, *J* = 11.2 Hz, 1 H), 4.283 (t, *J* = 6 Hz, 2 H), 4.196 (t, 5.6 Hz, 2 H), 2.772 (m, 4 H), 2.396‐2.307 (m, 5 H), 1.630 (t, *J* = 7.2 Hz, 2 H), 1.274 (s, 28 H), 0.901 (t, *J* = 6.4 Hz, 3 H); ESI‐MS (m/z): 440.3946 [M+H]^+^.

### The Synthesis of 2D‐Lipid and 3D‐Lipid—Synthesis of 3D‐Lipid

A mixture of 0.132 g ACR‐A_0_C_18_ (0.3 mmol) and 0.240 g CsA (0.2 mmol) were dissolved into 5.0 mL THF, and stirred at 85 °C for 4 h. Then, THF was removed under reduced pressure and the crude product was purified by column chromatography (DCM/MeOH = 150/1 → 20/1) to obtain 0.078 g 3D‐lipid (24.4% yield) as a brown solid powder. ^1^H NMR (400 MHz, CDCl_3_‐*d6*): *δ* (ppm) 7.518‐7.362 (m, 1 H), 7.089‐6.906 (m, 3 H), 5.509‐5.488 (m, 2 H), 5.418‐5.295 (m, 2 H), 5.118‐4.975 (m, 2 H), 4.869‐4.770 (m, 3 H), 4.395‐4.198 (m, 7 H), 3.838‐3.695 (m, 3 H), 3.335‐3.169 (m, 2 H), 3.136‐2.797 (m, 18 H), 2.667 (s, 3 H), 2.366‐2.305 (m, 10 H), 1.732‐1.601 (m, 14 H), 1.431 (m, 11 H), 1.246 (s, 28 H), 0.931‐0.873 (m, 45 H); ESI‐MS (m/z): 1600.1836 [M+H]^+^.

### Preparation and Characterization of LNPs

The microfluidic technology was used to prepare the siRNA‐loaded LNPs. siRNA was dissolved in citric acid/sodium citrate buffer (10 mmol L^−1^, pH = 4.0, RNase free) to obtain the solution A. The lipid mixture (Tables S1 and S2, Supporting Information) was prepared in ethanol to obtain the solution B. Solution A and solution B were rapidly mixed using a microfluidic chip at an aqueous/ethanol volumetric ratio of 3/1 and a flow rate of 4.0 mL min^−1^. The resulting mixture was dialyzed (8000 – 14 000 Da) in PBS (RNase free) overnight. The siRNA encapsulation efficiency of LNPs was measured using a Quant‐iT Ribogreen assay kit (ThermoFisher, USA).

The particle size and zeta potential of siRNA‐loaded LNPs were detected by dynamic light scattering (DLS, Malvern Zetasizer Nano ZS, Malvern, UK). The prepared LNPs were dropped onto a copper and then dried at 37 °C overnight. The morphology of LNPs was observed using scan electron microscope (SEM, JSM‐7900F, Japan) under transmission mode. The shear resistance of siRNA‐loaded LNPs was reflected by the △size and △PDI of LNPs after nebulization using a nebulizer. The storage stability of LNPs was reflected by the size stability within 7 days. The dilution stability of LNPs was reflected by the size stability at different dilution ratio by PBS.

### Gel Retardation Assay

To evaluate the protective effect of LNPs on siRNA, the LNPs were incubated with 10% FBS at 37 °C. At predetermined time points, the samples were collected and mixed with 6 × loading buffer and Triton X‐100. And then, the electrophoresis was carried out on a 1% agarose gel containing 0.1 Gelred at 80 mV for 3 min, subsequently 100 mV for 15 min. The resulting gel was observed on an Amersham Imager 600 system (GE Healthcare Life Sciences, USA) under UV‐illumination. Free siRNA was used as the control.

### pH‐Responsive Release of siRNA from LNPs

The prepared Cy5‐siRNA‐loaded LNPs were dialyzed (20 000 Da) in PBS (V_LNPs_/V_PBS_ = 1/20) at different pH (7.4 and 5.0). At predetermined time points, the fluorescence intensity of released Cy5‐siRNA was measured at an excitation wavelength of 650 nm and an emission wavelength of 670 nm using a microplate reader (Thermo scientific, USA). Cumulative siRNA release rate was calculated as A_sample_/A_total_ × 100%. A_total_ represents the fluorescence intensity of total Cy5‐siRNA completely released from LNPs.

### LUC Assay

A549‐LUC cells (5 × 10^3^ cells per well) were seeded into 96‐well plates and incubated with 0.2 mL F12K medium containing siLUC‐loaded LNPs (after nebulization using a nebulizer) for 24 h. Then the medium was removed and the LUC expression level was detected using a Firefly Glo Luciferase Reporter Gene Assay Kit (Yeasen, Shanghai, China). The final siLUC concentration was fixed at 100 nmol L^−1^.

### The Docking and Molecular Dynamics Simulation

The Discovery Studio software was used to build the structures of 2D‐lipid and 3D‐lipid. Then 5000 steps minimization was performed to obtain the stable structures. The docking process of two 3D‐lipid molecules (complex 1), as well as cyclophilin D (CypD, PDB ID: 2Z6 W) and 3D‐lipid (complex 2) were carried out by using HDOCK server.^[^ ^42^ ^]^

The simulation was performed with the AMBER 16 molecular simulation package. To obtain molecular mechanical parameters for 2D‐lipid and modified residues of 3D‐lipid, ab initio quantum chemical methods were employed using the Gaussian 16 program. The geometry was fully optimized, and then the electrostatic potentials around them were determined at the B3LYP/6‐31G* level of theory. The RESP strategy was used to obtain the partial atomic charges.

The starting structures of 2D‐lipid, 3D‐lipid, complex 1, and complex 2 obtained by docking were solvated in TIP3P water using an octahedral box, which extended 8 Å away from any solute atom. MD simulation was carried out by using PMEMD module of AMBER 16. The calculations began with 500 steps of steepest descent followed by 500 steps of conjugate gradient minimization with a large constraint of 500 kcal mol^−1^ Å^−2^ on the atoms of the studied systems. Then 1000 steps of steepest descent followed by 4000 steps of conjugate gradient minimization with no restraint on all atoms were performed. Subsequently, after 200 ps of MD, during which the temperature was slowly raised from 0 to 300 K with weak (5 kcal mol^−1^ Å^−2^) restraint on the studied systems, the final unrestrained production simulations of 40.0 ns were carried out at constant pressure (1 atm) and temperature (300 K). In the entire simulation, SHAKE was applied to all hydrogen atoms. Periodic boundary conditions with minimum image conventions were applied to calculate the non‐bonded interactions. A cutoff of 10 Å was used for the Lennard–Jones interactions. The final conformations of the studied systems used for discussion were produced from the 1000 steps of minimized averaged structure of the last 20.0 ns of MD.

The binding energy between two 3D‐lipid molecules were calculated using MM_PBSA scripts supplied by AMBER 16.^[^ ^43^ ^]^ A single‐trajectory approach was used to extract 200 snapshots for the MM_PBSA calculation from each of the last 20 ns trajectories at 100 ps intervals.

### FRET Assay

For detecting lipid fusion rate, anionic endosomal mimics loading FRET probes (DOPS/DOPC/DOPE/NBD‐PE/Rho‐PE = 25/25/50/1/1, mol%) and LNPs (ionizable lipid/DSPC/Chol/DMG‐PEG2000 = 30/35/33.5/1.5, mol%) were prepared by thin‐film rehydration method.^[^ ^27^ ^]^ The total lipid concentrations of anionic endosomal mimics and LNPs were 5.0 mmol L^−1^ and 1.5 mmol L^−1^, respectively. The anionic endosomal mimics and LNPs were mixed in equal volume, and 0.1 mmol L^−1^ HCl solution was used to adjust the pH of the mixture to 6.2 and 5.0. After incubating at 37 °C for 10 min, the fluorescence intensity of the sample (F_SPL_) was measured using a microplate reader (Thermo scientific, USA) at the excitation wavelength of 465 nm and emission wavelength of 520 nm. The fluorescence intensity of the mixture at pH = 7.4 was set as negative control (F_min_). The fluorescence intensity of the mixture incubated with Triton X‐100 solutions was set as positive control (F_max_). The lipid fusion rate was calculated as (F_SPL_ – F_min_)/(F_max_ – F_min_) × 100%.

### In Vitro Cellular Uptake

For flow cytometry, A549 cells (2 × 10^5^ cells per well) were seeded into 12‐well plates and incubated for 24 h. Then the cells were treated with BLM (20 µg mL^−1^) and Cy5‐siRNA‐loaded LNPs for 6 h. Subsequently, the cells were digested using trypsin‐EDTA and collected by centrifugation (2000 rpm) for 5 min. After resuspension with PBS, the intracellular fluorescence intensity was detected by a FACS Calibur flow cytometry (Becton Dickinson, San Jose, CA, USA). The final Cy5‐siRNA concentration was all fixed at 100 nmol L^−1^.

For CLSM imaging, A549 cells (4 × 10^5^ cells per well) were seeded in confocal dishes (20 mm) and incubated for 24 h. Then the cells were treated with BLM (20 µg mL^−1^) and Cy5‐siRNA‐loaded LNPs for 6 h. After incubation, the A549 cells were fixed by 4% paraformaldehyde for 15 min and stained using Dil (10 µmol L^−1^) for 20 min. And then, the cells were washed by PBS, treated with 1.0 mL anti‐fluorescence attenuation encapsulation agent (including DAPI) and visualized under a confocal fluorescence microscope (Nikon, Japan). The final Cy5‐siRNA concentration was fixed at 100 nmol L^−1^. The final concentrations of 3D‐lipid and 2D‐lipid were fixed at 25 µmol L^−1^.

### Lysosomal Escape

A549 cells (4 × 10^5^ cells per well) were seeded in confocal dishes (20 mm) and incubated for 24 h. Then the cells were treated with BLM (20 µg mL^−1^) and Cy5‐siRNA‐loaded NPs for 6 h. After incubation, the A549 cells were fixed by 4% paraformaldehyde for 15 min and stained by LysoTracker Red DND‐99 (200 nmol L^−1^) for 30 min. And then, the cells were washed by PBS, treated with 1.0 mL anti‐fluorescence attenuation encapsulation agent (including DAPI) and visualized under a confocal fluorescence microscope (Nikon, Japan). The final Cy5‐siRNA concentration was fixed at 100 nmol L^−1^. The final concentrations of 3D‐lipid and 2D‐lipid were fixed at 25 µmol L^−1^.

### *P* Value Calculation

The *P* values of 3D‐lipid and 2D‐lipid were calculated as: *P* = *V*/(*lc* × *a*), where *V* is the volume of the lipid, *lc* is the critical tail length and *a* is the head area of the lipid. To calculate the *P* values of 3D‐lipid and 2D‐lipid, the Discovery Studio software was used to build the structures of 2D‐lipid and 3D‐lipid. Then 5000 steps minimization was performed to obtain the energy‐minimized structures. the *a* was measured as the cross section area of head group, which was a spherical structure with a radius equal to the sum of C─N and C─H bond lengths. The *lc* was measured as the vertical distance between the center of mass (COM) of tertiary amine group and the two terminal methyl groups. The *V* was measured using an approximate method named Atomic and Bond Contributions of van der Waals volume (VABC): *V*_vdW_ = Σ_all atom contributions_ – 5.92*N_B_* – 14.7*R_A_* – 3.8*R_NA_*, where *N_B_* is the number of bonds, *R_A_* is the number of aromatic rings, and *R_NA_* is the number of nonaromatic rings.^[^ ^44^ ^]^

### Detection of mRNA Levels

For cellular experiments, A549 cells (4 × 10^5^ cells per well) were seeded into 6‐well plates and incubated for 24 h. Then the cells were treated with BLM (20 µg mL^−1^) and different formulations for 24 h. Subsequently, TRIZOL reagent (Invitrogen, USA) was used to extract the total RNA of cells. The Reverse Transcription Kit GoScript Reverse Transcription System (#A5001, Promega, USA) was used to reversely transcribe the RNA. And then GoTaqqPCR Master Mix (#A6002, Promega, USA) was used to amplify the cDNA. The mRNA levels of the target gene were detected by a Real‐Time PCR amplifier (MX3005P, Stratagene, USA). The GAPDH was used as endogenous control. The nanoformulations were pre‐treated with nebulizer. The final siRNA concentration was fixed at 100 nmol L^−1^. The final concentrations of CsA, 3D‐lipid and 2D‐lipid were fixed at 25 µmol L^−1^.

For the lung tissue analysis, the human lung tissues or animal lung tissues were ground in TRIZOL reagent (Invitrogen, USA) and the subsequent operations were performed as the same as described above. The GAPDH was used as endogenous control for the analysis of human lung tissues and the *β*‐Actin was used as endogenous control for the analysis of animal lung tissues.

### Western Blotting Assay

A549 cells (4 × 10^5^ cells per well) were seeded into 6‐well plates and incubated for 24 h. Then the cells were treated with BLM (20 µg mL^−1^) and different formulations for 24 h. Subsequently, the cells were lysed in Radio‐Immunoprecipitation Assay (RIPA) buffer containing 1% phenyl methane sulfonyl fluoride (PMSF) for 30 min at 4 °C. The lysates were collected and centrifuged (12 000 rpm) at 4 °C for 15 min. The supernatant was collected and the total protein was quantified using BCA protein assay kit (Solarbio, Beijing, China). And then, protein samples (40 µg) were loaded and separated by 10% sodium dodecyl sulfate polyacrylamide gel electrophoresis (SDS‐PAGE) and transferred to a Polyvinylidene Fluoride (PVDF) membrane. After blocking with 5% nonfat milk in TBST for 2 h, the membranes were first incubated with the primary antibodies overnight at 4 °C, and then incubated with a horseradish peroxidase (HRP)‐labeled secondary antibody at room temperature for 2 h. The protein bonds were visualized by enhanced chemiluminescence reagent (millipore, USA) and imaged using Amersham Imager 600 system (GE Healthcare Life sciences, USA). The *β*‐Tubulin was used as endogenous control. The nanoformulations were pre‐treated with nebulizer. The final siRNA concentration was fixed at 100 nmol L^−1^. The final concentrations of CsA, 3D‐lipid and 2D‐lipid were fixed at 25 µmol L^−1^.

### Detection of mPTP Opening Level

For flow cytometry, A549 cells (2 × 10^5^ cells per well) were seeded into 12‐well plates and incubated for 24 h. Then the cells were treated with BLM (20 µg mL^−1^) and different formulations for 24 h. Subsequently, the cells were digested using trypsin‐EDTA and collected by centrifugation (2000 rpm) for 5.0 min. The cells were stained using calcein‐AM and treated with CoCl_2_ for 15 min. The intracellular fluorescence intensity was detected by a FACS Calibur flow cytometry (Becton Dickinson, San Jose, CA, USA).

For CLSM imaging, A549 cells (4 × 10^5^ cells per well) were seeded in confocal dishes (20 mm) and incubated for 24 h. Then the cells were treated with BLM (20 µg mL^−1^) and different formulations for 24 h. Subsequently, the cells were stained using calcein‐AM and treated with CoCl_2_ for 15 min. The cells were washed by PBS and fixed by 4% paraformaldehyde for 15 min. And then, the cells were washed by PBS, treated with 1.0 mL antifade mounting medium (with DAPI) and visualized under a confocal fluorescence microscope (Nikon, Japan). The nanoformulations were pre‐treated with nebulizer. The final siRNA concentration was fixed at 100 nmol L^−1^. The final concentrations of CsA, 3D‐lipid and 2D‐lipid were fixed at 25 µmol L^−1^.

### Detection of mtROS Level

For flow cytometry, A549 cells (2 × 10^5^ cells per well) were seeded into 12‐well plates and incubated for 24 h. Then the cells were treated with BLM (20 µg mL^−1^) and different formulations for 24 h. Subsequently, the cells were digested using trypsin‐EDTA and collected by centrifugation (2000 rpm) for 5 min. The cells were stained using MitoSOX Red (5 µmol L^−1^) for 20 min. The intracellular fluorescence intensity was detected by a FACS Calibur flow cytometry (Becton Dickinson, San Jose, CA, USA).

For CLSM imaging, A549 cells (4 × 10^5^ cells per well) were seeded in confocal dishes (20 mm) and incubated for 24 h. Then the cells were treated with BLM (20 µg mL^−1^) and different formulations for 24 h. Subsequently, the cells were stained by MitoSOX Red (5 µmol L^−1^) for 20 min. The cells were washed by PBS and fixed by 4% paraformaldehyde for 15 min. And then, the cells were washed by PBS, treated with 1.0 mL antifade mounting medium (with DAPI) and visualized under a confocal fluorescence microscope (Nikon, Japan). The nanoformulations were pre‐treated with nebulizer. The final siRNA concentration was fixed at 100 nmol L^−1^. The final concentrations of CsA, 3D‐lipid, and 2D‐lipid were fixed at 25 µmol L^−1^.

### Detection of Mitochondrial Membrane Potential Level

For flow cytometry, A549 cells (2 × 10^5^ cells per well) were seeded into 12‐well plates and incubated for 24 h. Then the cells were treated with BLM (20 µg mL^−1^) and different formulations for 24 h. Subsequently, the cells were digested using trypsin‐EDTA and collected by centrifugation (2000 rpm) for 5 min. The cells were stained using JC‐1 (10 µmol L^−1^) for 20 min. The intracellular fluorescence intensity of JC‐1 polymer and JC‐1 monomer was detected by a FACS Calibur flow cytometry (Becton Dickinson, San Jose, CA, USA).

For CLSM imaging, A549 cells (4 × 10^5^ cells per well) were seeded in confocal dishes (20 mm) and incubated for 24 h. Then the cells were treated with BLM (20 µg mL^−1^) and different formulations for 24 h. Subsequently, the cells were stained by JC‐1 (10 µmol L^−1^) for 20 min. The cells were washed by PBS and fixed by 4% paraformaldehyde for 15 min. And then, the stained cells were washed by PBS, treated with 1.0 mL antifade mounting medium (with DAPI) and visualized under a confocal fluorescence microscope (Nikon, Japan). The nanoformulations were pre‐treated with nebulizer. The final siRNA concentration was fixed at 100 nmol L^−1^. The final concentrations of CsA, 3D‐lipid and 2D‐lipid were fixed at 25 µmol L^−1^.

### Detection of Intracellular ROS Level

For flow cytometry, A549 cells (2 × 10^5^ cells per well) were seeded into 12‐well plates and incubated for 24 h. Then the cells were treated with BLM (20 µg mL^−1^) and different formulations for 24 h. Subsequently, the cells were digested using trypsin‐EDTA and collected by centrifugation (2000 rpm) for 5 min. The cells were stained using DCFH‐DA (10 µmol L^−1^) for 20 min. The intracellular fluorescence intensity was detected by a FACS Calibur flow cytometry (Becton Dickinson, San Jose, CA, USA).

For CLSM imaging, A549 cells (4 × 10^5^ cells per well) were seeded in confocal dishes (20 mm) and incubated for 24 h. Then the cells were treated with BLM (20 µg mL^−1^) and different formulations for 24 h. The cells were stained by DCFH‐DA (10 µmol L^−1^) for 20 min and MitoTracker Red (10 µmol L^−1^) for 20 min. The cells were washed by PBS and fixed by 4% paraformaldehyde for 15 min. And then, the cells were washed by PBS, treated with 1.0 mL antifade mounting medium (with DAPI) and visualized under a confocal fluorescence microscope (Nikon, Japan). The nanoformulations were pre‐treated with nebulizer. The final siRNA concentration was fixed at 100 nmol L^−1^. The final concentrations of CsA, 3D‐lipid and 2D‐lipid were fixed at 25 µmol L^−1^.

### Detection of Intracellular NO Level

For flow cytometry, A549 cells (2 × 10^5^ cells per well) were seeded into 12‐well plates and incubated for 24 h. Then the cells were treated with BLM (20 µg mL^−1^) and different formulations for 24 h. The cells were stained using DAF‐FM DA (5 µmol L^−1^) for 20 min. The intracellular fluorescence intensity was detected by a FACS Calibur flow cytometry (Becton Dickinson, San Jose, CA, USA).

For CLSM imaging, A549 cells (4 × 10^5^ cells per well) were seeded in confocal dishes (20 mm) and incubated for 24 h. Then the cells were treated with BLM (20 µg mL^−1^) and different formulations for 24 h. Subsequently, the cells were stained by DAF‐FM DA (5 µmol L^−1^) for 20 min and MitoTracker Red (10 µmol L^−1^) for 20 min. The cells were washed by PBS and fixed by 4% paraformaldehyde for 15 min. And then, the cells were washed by PBS, treated with 1.0 mL antifade mounting medium (with DAPI) and visualized under a confocal fluorescence microscope (Nikon, Japan). The nanoformulations were pre‐treated with nebulizer. The final siRNA concentration was fixed at 100 nmol L^−1^. The final concentrations of CsA, 3D‐lipid and 2D‐lipid were fixed at 25 µmol L^−1^.

### Cytotoxicity Assay

CCK‐8 assay was used to evaluate the cytotoxicity of siMCU‐loaded LNPs. A549 cells (8 × 10^3^ cells per well) were seeded into the 96‐well plates and incubated for 24 h. Then the cells were treated with different LNPs for 24 h. The cells treated with PBS were used as the negative control. Subsequently, CCK‐8 (10 µL) solution was added into each well, and then the cells were cultured for another 2 h. The absorbance was measured at 450 nm using a microplate reader (Thermo scientific, USA). The cell viability was calculated as A_sample_/A_control_. The final siRNA concentration was fixed at 100 nmol L^−1^. The final concentrations of 3D‐lipid and 2D‐lipid were fixed at 25 µmol L^−1^.

### Detection of p‐NF‐*κ*B p65/NF‐*κ*B p65 Ratio

A549 cells (4 × 10^5^ cells per well) were seeded into 6‐well plates and incubated for 24 h. Then the cells were treated with BLM (20 µg mL^−1^) and different formulations for 24 h. Subsequently, the cells were lysed in 150 µL RIPA lysis buffer containing 1% proteinase inhibitor cocktail for 30 min. After centrifugation (12 000 rpm) for 10 min, the supernatant was collected and used to detect the expression level of p‐NF‐*κ*B p65 and NF‐*κ*B p65 using a human p‐NF‐*κ*B p65 and NF‐*κ*B p65 ELISA kits according to the manufacturer's protocol (protein levels had been used to normalize the p‐NF‐*κ*B p65 and NF‐*κ*B p65 levels). The absorbance was measured at 450 nm using a microplate reader (Thermo scientific, USA). The standard curves were established for calculating the expression levels of p‐NF‐*κ*B p65 and NF‐*κ*B p65. The nanoformulations were pre‐treated with nebulizer. The final siRNA concentration was fixed at 100 nmol L^−1^. The final concentrations of CsA, 3D‐lipid and 2D‐lipid were fixed at 25 µmol L^−1^.

### Detection of Nuclear Translocation of NF‐*κ*B p65

A549 cells (4 × 10^5^ cells per well) were seeded in confocal dishes (20 mm) and incubated for 24 h. Then the cells were treated with BLM (20 µg mL^−1^) and different formulations for 24 h. The cells were fixed by 4% paraformaldehyde for 15 min, and then treated with an immunostaining blocking solution for 1 h. The cells were washed by PBS, stained with NF‐*κ*B p65 mouse monoclonal antibody for 1 h and incubated with anti‐mouse Cy3 antibody for another 1 h. And then, the cells were washed by PBS, treated with 1.0 mL antifade mounting medium (with DAPI) and visualized under a confocal fluorescence microscope (Nikon, Japan). The nanoformulations were pre‐treated with nebulizer. The final siRNA concentration was fixed at 100 nmol L^−1^. The final concentrations of CsA, 3D‐lipid, and 2D‐lipid were fixed at 25 µmol L^−1^.

### Establishment of 3D Multicellular Lung Spheroid Models

The 3D multicellular lung spheroid models were prepared based on a previously reported method with several modification.^[^ ^37^ ^]^ Briefly, A549 cells and Hs888lu cells were seeded in an ultra‐low attachment 96‐well plate with a A549/Hs888lu ratio of 2/1. Their morphology and size were monitored by bright field microscopy during the incubation. Fluorescence imaging was used to distinguish different cell types. A549 cells were stained by Vybrant DiO, and Hs888lu cells were stained by Vybrant CM‐DiI.

### Immunohistochemistry and Immunofluorescence Analysis on 3D Multicellular Lung Spheroid Models

After 4‐day incubation, the 3D multicellular lung spheroid models were treated with BLM (20 µg mL^−1^) and different formulations for 48 h. Subsequently, the collected 3D multicellular lung spheroid models were fixed in a 4% paraformaldehyde solution. The samples were embedded in paraffin and cut into 5 µm‐thick sections. And then the sections were sequentially treated with xylene, 95% alcohol, 85% alcohol, and 75% alcohol for 5 min, and washed by PBS after each treatment. Subsequently, the sections were treated with primary antibodies overnight at 4 °C and then biotinylated secondary antibody for 1 h. After washing by PBS, the sections were treated with DAB and then stopped in distilled water. After re‐staining with hematoxylin, a pathological section scanner (Buchi) was used to observe the stained sections. Immunofluorescence colocalization analyses were performed using the primary antibody and secondary antibody. The stained sections were visualized using a standing fluorescence microscope (Nikon Eclipse C1, Nikon, Japan). The nanoformulations were pre‐treated with nebulizer. The final siRNA concentration was fixed at 100 nmol L^−1^. The final concentrations of 3D‐lipid and 2D‐lipid were fixed at 25 µmol L^−1^.

### Biodistribution

The C57BL/6J mice (male, 6–8 weeks) were administrated with Cy5‐siRNA‐loaded NPs through pulmonary administration using a nebulizer. At pre‐determined time intervals, mice were sacrificed to harvest major organs including lung, heart, liver, spleen, stomach, and kidneys. The fluorescent images of major organs were observed using an in vivo imaging system (Imaging Station Maestro2, CRI, USA). The ROI was analyzed by Living Image software. The siRNA was administrated at a dose of 0.2 mg kg^−1^, while the 3D‐lipid and 2D‐lipid doses were 6.0 and 2.4 mg kg^−1^.

### Establishment of BLM‐Induced PF Mouse Models

The C57BL/6J mice (male, 6–8 weeks) were treated with BLM (1.0 mg kg^−1^) through pulmonary pathway using a nebulizer. One day after the treatment of BLM, different formulations (Saline, MC3/siMCU NPs, 2D‐LNP/siMCU NPs, 3D‐LNP/siNC NPs, 3D‐LNP/siMCU NPs, and Opt‐MC3/siMCU NPs) were administrated through pulmonary pathway using a nebulizer every seven days, four dosages in total. PFD was administrated daily via intragastric delivery as a positive control. The healthy mice were used as the normal group. On day 28 after the first administration, the mice were executed and the lung tissues were collected for subsequent analysis. The siRNA was administrated at a dose of 0.2 mg kg^−1^ per administration, while the 3D‐lipid and 2D‐lipid doses were 6.0 and 2.4 mg kg^−1^ per administration, respectively. The PFD was administrated at a dose of 0.2 mg kg^−1^.

### Histological Staining Analysis on Lung Tissues

The lung tissues were collected and fixed in a 4% paraformaldehyde solution. The lung samples were embedded in paraffin and cut into 6 µm‐thick sections. H&E staining was performed to evaluate the histopathology of lung tissues and Masson's trichrome staining was performed to evaluate the collagen deposition in lung tissues. Stained slides were observed by a pathological slide scanner (Buchi). The fibrotic ratio was calculated by quantifying the ratio of blue area in the Masson's trichrome section using Image J software.

### In Vivo Detection of MCU, Col‐I, Col‐III, *α*‐SMA, and TGF‐*β* Expression Levels

For examining the expression levels of Col‐I, Col‐III, *α*‐SMA, and TGF‐*β* in the lung tissues, the homogenates of the lung tissues were centrifuged (12 000 rpm) and the supernatants were collected for subsequent measurements using ELISA kits according to the manufacturer's protocols (protein levels had been used to normalize the MCU, Col‐I, Col‐III, *α*‐SMA, and TGF‐*β* levels). The absorbance was measured at 450 nm using a microplate reader (Thermo scientific, USA). The standard curves were established for calculating the MCU, Col‐I, Col‐III, *α*‐SMA, and TGF‐*β* levels in samples.

### In Vivo Detection of HYP Level

A HYP detection kit was used to detect the HYP level in lung tissues. According to the manufacturer's protocol, the lung tissues were collected and digested at 37 °C for 3 h. And then the samples were centrifuged (12 000 rpm) to obtain supernatants. A series of reaction agents were subsequently added into the supernatants for the chromogenic reactions following the manufacturer's instructions. The absorbance was measured at 560 nm using a microplate reader (Thermo scientific, USA). The standard curve was established for calculating the HYP levels in samples.

### In Vivo Detection of MDA Level

An MDA detection kit was used to detect the MDA level in lung tissues. According to the manufacturer's protocol, the lung tissues were collected and digested at 37 °C for 3 h. And then the samples were centrifuged (12 000 rpm) to obtain supernatants. A series of reaction agents were subsequently added into the supernatants for the chromogenic reactions following the manufacturer's instructions. The reaction solution was transferred into 96‐well plate, and the absorbance was measured at 532 and 600 nm using a microplate reader (Thermo scientific, USA). The MDA level (nmol) was calculated as 15.48 × (A_532_ – A_600_).

### In Vivo Detection of H_2_O_2_ Level

A H_2_O_2_ detection kit was used to detect the H_2_O_2_ level in lung tissues. According to the manufacturer's protocol, the lung tissues were collected and digested at 37 °C for 3 h. And then the samples were centrifuged (12 000 rpm) to obtain supernatants. A series of reaction agents were subsequently added into the supernatants for the chromogenic reactions following the manufacturer's instructions. The reaction solution was transferred into 96‐well plate, and the absorbance was measured at 415 nm. The H_2_O_2_ level (mmol L^−1^) was calculated as (A_415_ + 0.08)/0.031.

### In Vivo Detection of NO Level

For examining the NO level in lung tissues, the homogenates of the lung tissues were centrifuged (12 000 rpm) and the supernatants were collected for subsequent measurements using a Griess kit according to the manufacturer's protocols. The absorbance was measured at 540 nm using a microplate reader (Thermo scientific, USA). The standard curves were established for calculating the NO level in samples.

### Immunohistochemistry and Immunofluorescence Colocalization Analyses on Lung Tissues

The lung tissues were collected and fixed in a 4% paraformaldehyde solution. The lung samples were embedded in paraffin and cut into 5 µm‐thick sections. And then the sections were sequentially treated with xylene, 95% alcohol, 85% alcohol, and 75% alcohol for 5 min, and washed by PBS after each treatment. Subsequently, the sections were treated with primary antibodies overnight at 4 °C and then biotinylated secondary antibody for 1 h. After washing by PBS, the sections were treated with DAB and then stopped in distilled water. After re‐staining with hematoxylin, a pathological section scanner (Buchi) was used to observe the stained sections. Immunofluorescence colocalization analyses were performed using the primary antibody and secondary antibody. The stained sections were visualized using a standing fluorescence microscope (Nikon Eclipse C1, Nikon, Japan).

### In Vivo Biosafety Assay

The major organs (liver, brain, heart, spleen, and kidneys) were collected and fixed in a 4% paraformaldehyde solution. And then the sections were stained with H&E and observed by a pathological slide scanner (Buchi). For hematological assessments, blood samples were collected to detect the levels of RBC, HGB, and PLT.

### Statistical Analysis

The mean ± standard deviation (SD) was determined for all the treatment groups. Unpaired student's t‐test (two‐tailed) was used for two‐group comparison and one‐way ANOVA followed by Tukey test was applied for multiple‐group statistical analysis. All the statistical analyses were performed by using GraphPad Prism Software (Version 8.0, GraphPad Software, San Diego, CA). The difference between two groups was considered statistically significant for *P < 0.05, **P < 0.01, and ***P < 0.001.

## Conflict of Interest

The authors declare no conflict of interest.

## Supporting information



Supporting Information

## Data Availability

The data that support the findings of this study are available from the corresponding author upon reasonable request.
