# Supplementary material for: Double Braking Effects of Nanomedicine on Mitochondrial Permeability Transition Pore for Treating Idiopathic Pulmonary Fibrosis
Source: Adv Sci (Weinh). 2024 Oct 30;11(47):2405406. doi: 10.1002/advs.202405406 (PMC11653616; doi:10.1002/advs.202405406)
Supplement: Supplementary file 1 — Supporting Information [file ADVS-11-2405406-s001.docx]

Supporting Information

Double Braking Effects of Nanomedicine on Mitochondrial Permeability Transition Pore for Treating Idiopathic Pulmonary Fibrosis

*An Lu^a,†^, Zhiyi Xu^a,†^, Zhixia Zhao^b,†^, Yi Yan^a,†^, Linxia Jiang^a^, Jing Geng^c^, Hongwei Jin^a^, Xiangyu Wang^a^, Xiaoyan Liu^a^, Yuanjun Zhu^a^, Yujie Shi^a^, Lihong Liu^b^, Huaping Dai^c,*^, Jian-Cheng Wang^a,d,*^*

^a^Beijing Key Laboratory of Molecular Pharmaceutics and New Drug Delivery Systems, State Key Laboratory of Natural and Biomimetic Drugs, School of Pharmaceutical Sciences, Peking University, Beijing 100191, China.

^b^Department of Pharmacy, Clinical Trial Research Center, China-Japan Friendship Hospital, Beijing 100029, China.

^c^National Center for Respiratory Medicine；State Key Laboratory of Respiratory Health and Multimorbidity；National Clinical Research Center for Respiratory Diseases；Institute of Respiratory Medicine, Chinese Academy of Medical Sciences，Peking Union Medical College；Department of Pulmonary and Critical Care Medicine, Center of Respiratory Medicine, China-Japan Friendship Hospital, Beijing 100029, China.

^d^Laboratory of Innovative Formulations and Pharmaceutical Excipients, Peking University Ningbo Institute of Marine Medicine, Ningbo 315832, China.

***Corresponding authors.**

**Jian-Cheng Wang**, School of Pharmaceutical Sciences, Peking University, Beijing 100191, China. Email: wang-jc@bjmu.edu.cn.

**Huaping Dai**, Department of Pulmonary and Critical Care Medicine, China-Japan Friendship Hospital, Beijing 100029, China. Email: daihuaping@ccmu.edu.cn

**^†^**These authors made equal contributions to this work.


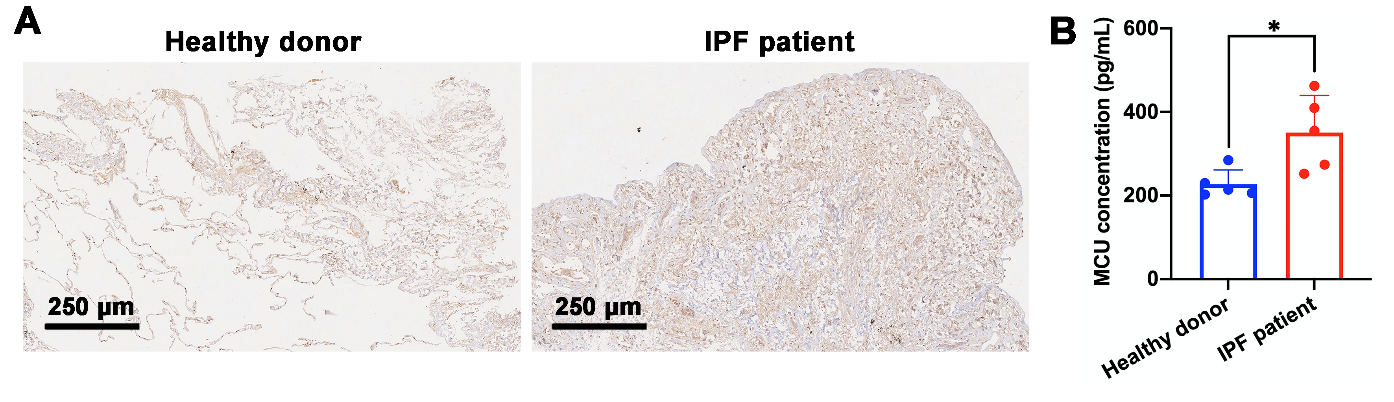


**Figure S1.** The expression level of MCU in IPF patients’ and healthy donors’ lung tissues. (A) Representative immunohistochemistry images to detect the expression level of MCU in IPF patients’ and healthy donors’ lung tissues. Scale bar, 250 μm; (B) The MCU expression levels in IPF patients’ and healthy donors’ lung tissues detected using an ELISA kit (n = 5). Data are represented as mean ± SD. **P* < 0.05, ***P* < 0.01, ****P* < 0.001.

**
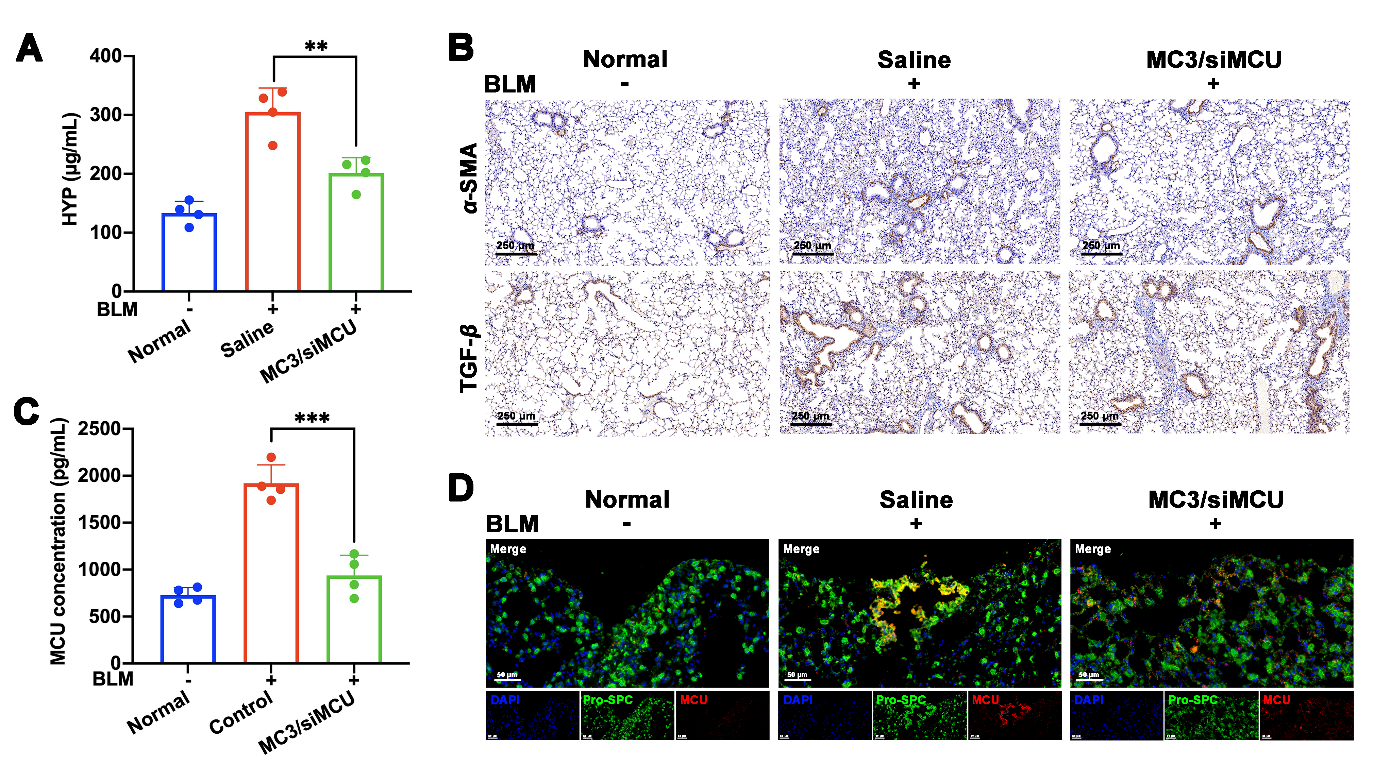
**

**Figure S2.** The in vivo effects of MC3/siMCU NPs on BLM-induced PF mouse models. (A) The HYP level in lung tissues isolated from mice detected using a HYP detection kit (n = 4); (B) Representative immunohistochemistry images to evaluate the expression levels of *α*-SMA and TGF-*β* in the lung tissue isolated from mice; (C) The MCU expression level in lung tissues isolated from mice detected by a ELISA kit (n = 4); (D) Representative immunofluorescence images of lung tissues isolated from mice to analyze the MCU (red) expression level in ACEIIs (using pro-SPC as a marker, green). The nucleus was stained with DAPI (blue). Scale bar, 250 μm. Data are represented as mean ± SD. **P* < 0.05, ***P* < 0.01, ****P* < 0.001.


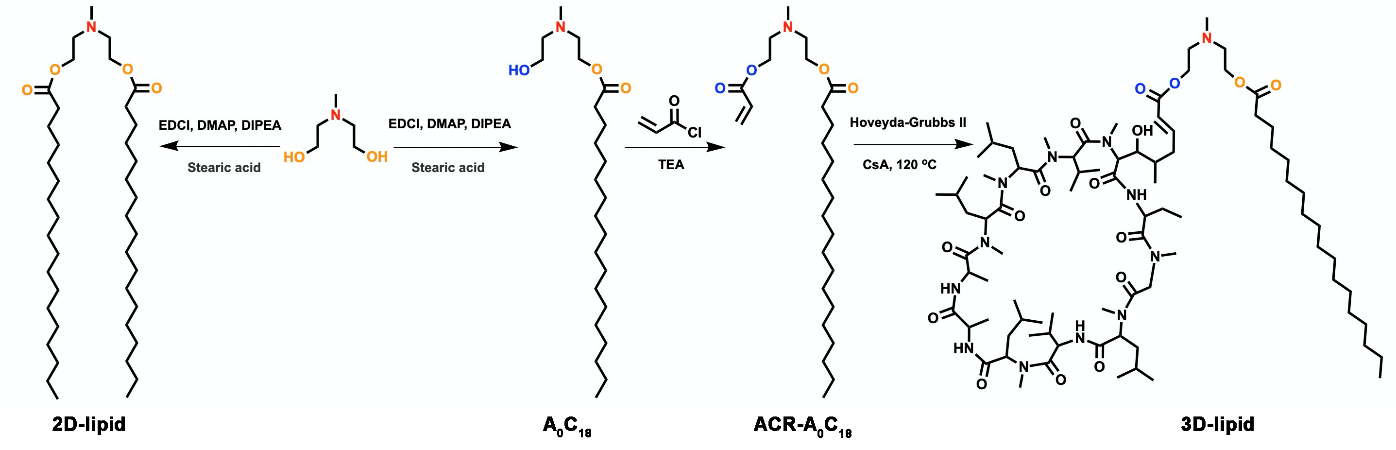


**Figure S3.** The synthetic route of the 3D-lipid and 2D-lipid.


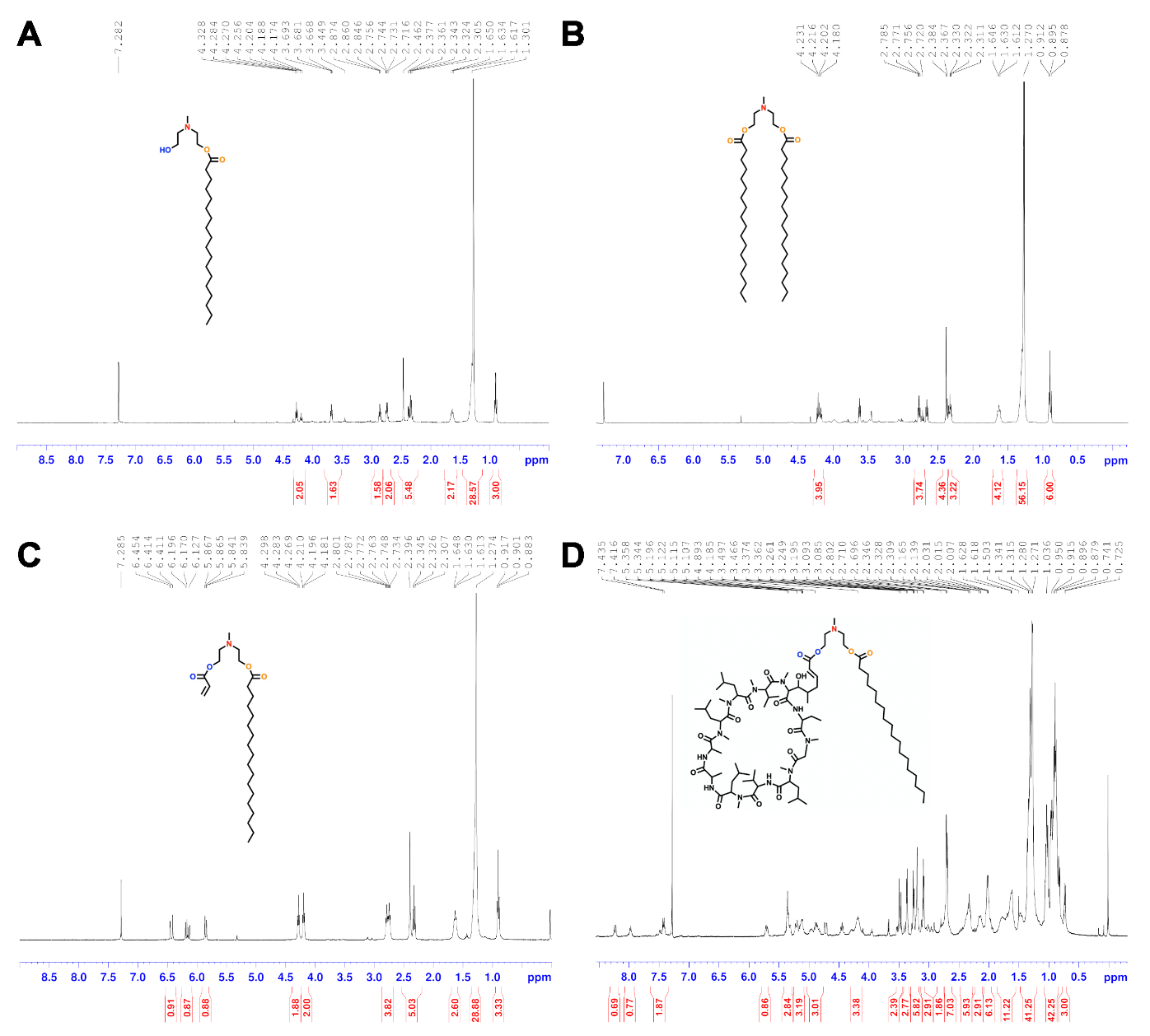


**Figure S4.** The ^1^H NMR of the A_0_C_18_ (A), 2D-lipid (B), ACR-A_0_C_18_ (C), and 3D-lipid (D).


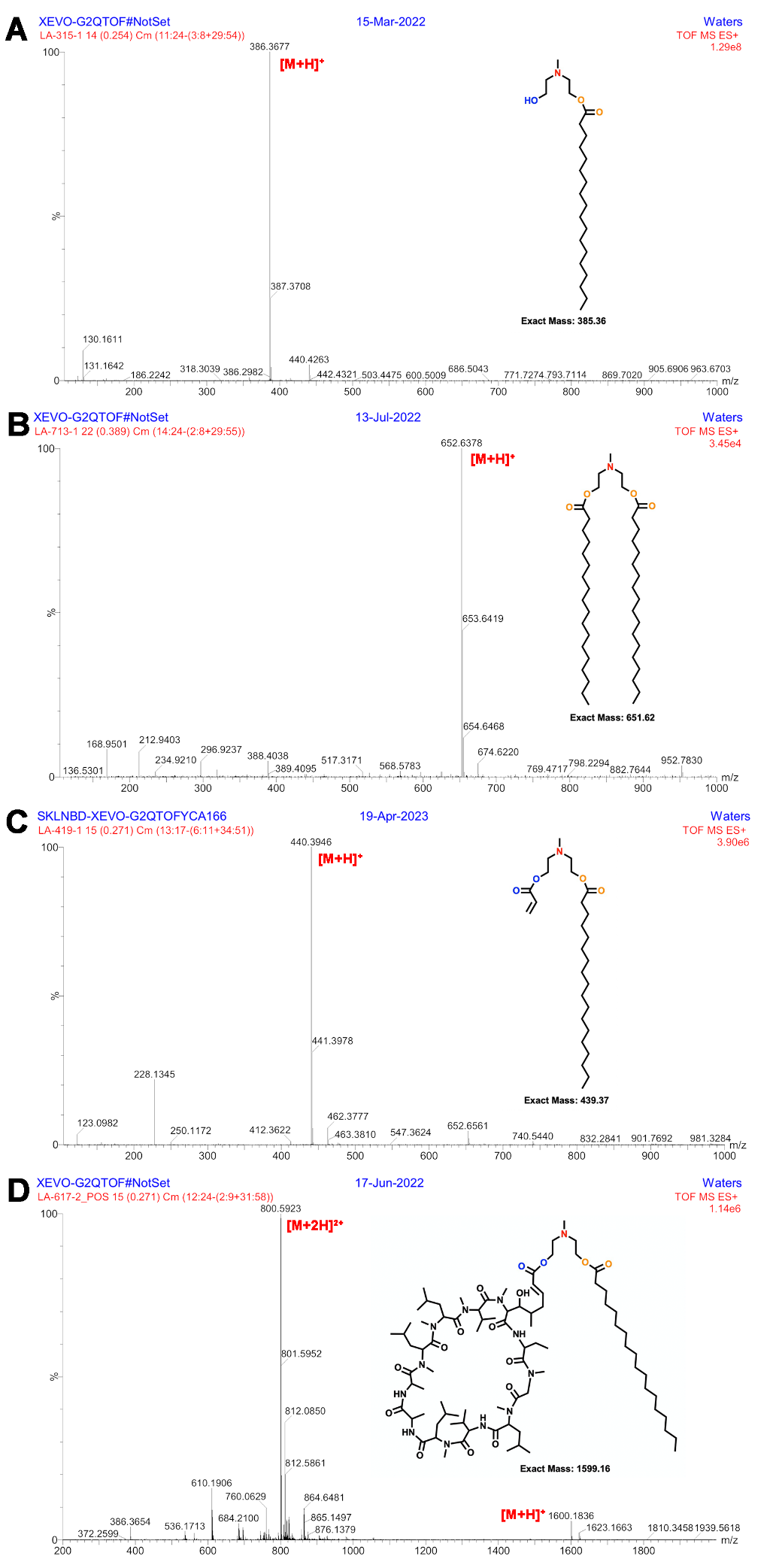


**Figure S5.** The ESI-MS of the A_0_C_18_ (A), 2D-lipid (B), ACR-A_0_C_18_ (C), and 3D-lipid (D).


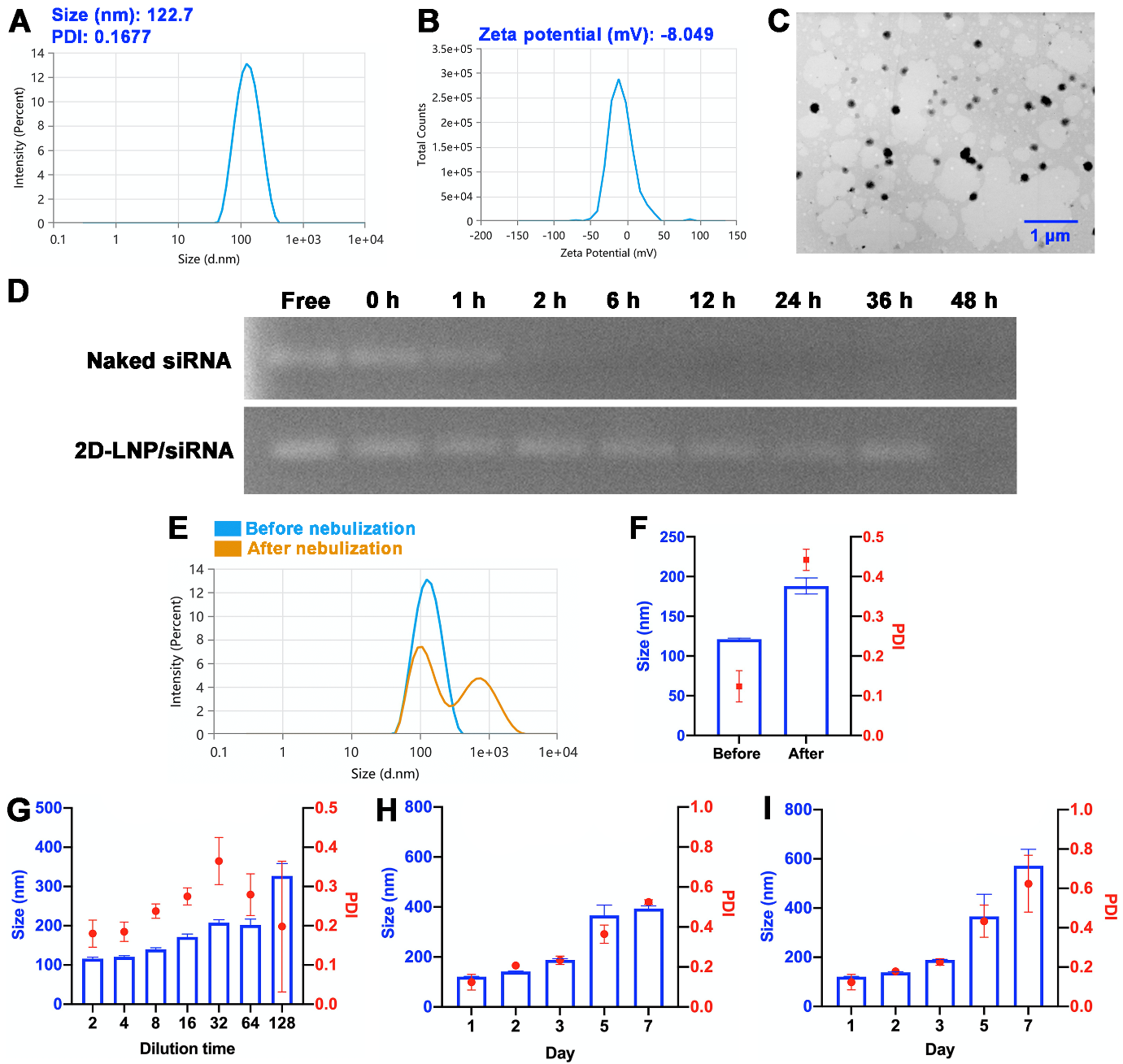


**Figure S6.** Characterization of 2D-LNP/siRNA NP. The particle size (A) and zeta potential (B) of 2D-LNP/siRNA NPs; (C) Representative STEM image of 2D-LNP/siRNA NPs. Scale bar, 1 μm; (D) The protective effect of 3D-LNP/siRNA NPs on siRNA after incubation with FBS for different time; (E) The size distribution of 3D-LNP/siRNA NPs before and after nebulization; (F) The particle size and PDI of 2D-LNP/siRNA NPs before and after nebulization (n = 3); The particle size and PDI of 2D-LNP/siRNA NPs after a 128-fold dilution (G), or 7-day storage at 4 ^o^C (H) and 20 ^o^C (I) (n = 3). Data are represented as mean ± SD. **P* < 0.05, ***P* < 0.01, *** *P* < 0.001.

**
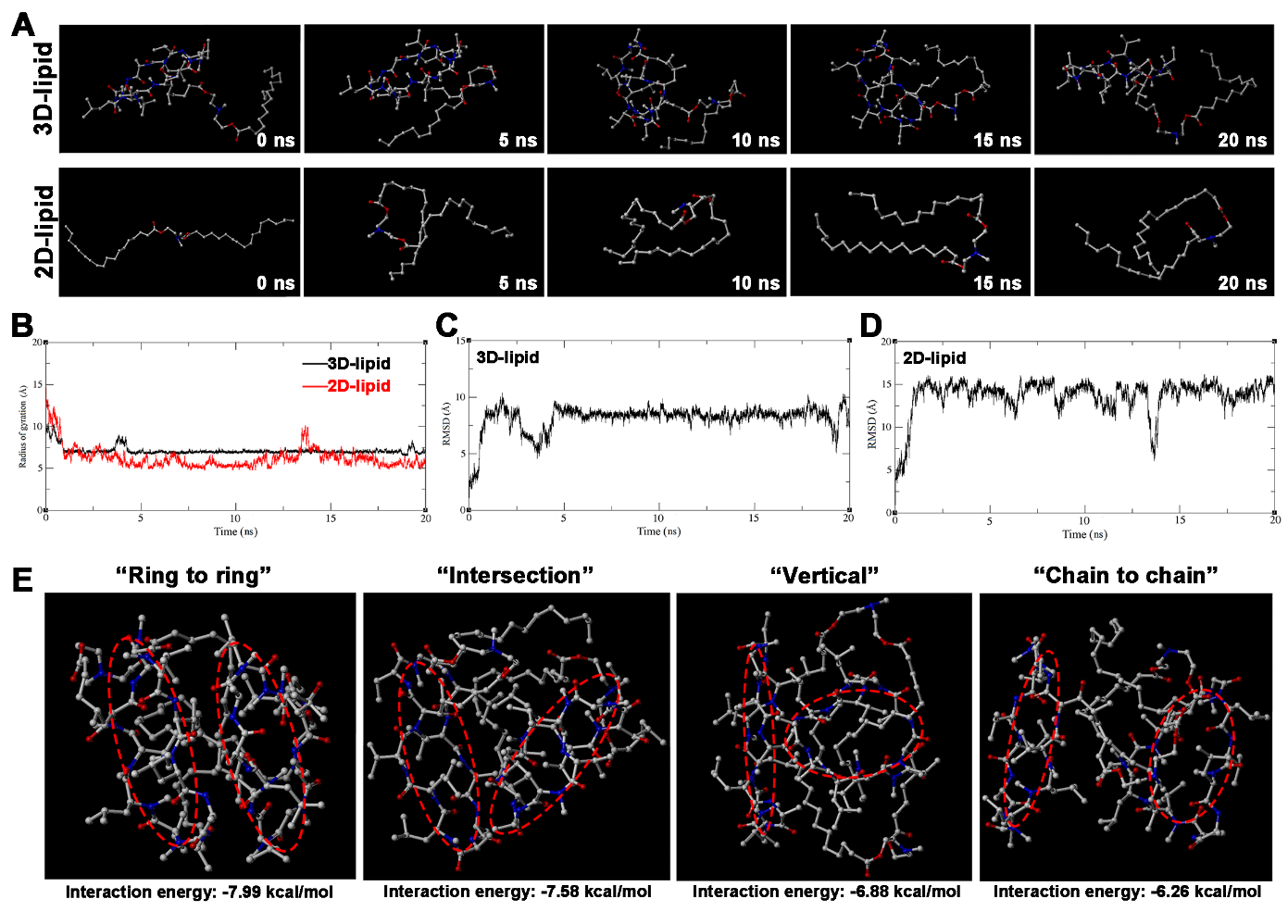
**

**Figure S7.** The molecular dynamics simulations of the 3D-lipid and 2D-lipid. (A) The conformational changes of the 3D-lipid and 2D-lipid in the molecular dynamics simulations; (B) Radius of gyration (*R_g_*) of the 3D-lipid and 2D-lipid for 20 ns in the molecular dynamics simulations; The root-mean-square deviation (RMSD) analyses of the 3D-lipid (C) and 2D-lipid (D) for 20 ns in the molecular dynamics simulations; (E) Four binging modes of two 3D-lipid molecules and their interaction energy in the molecular dynamics simulations.

**
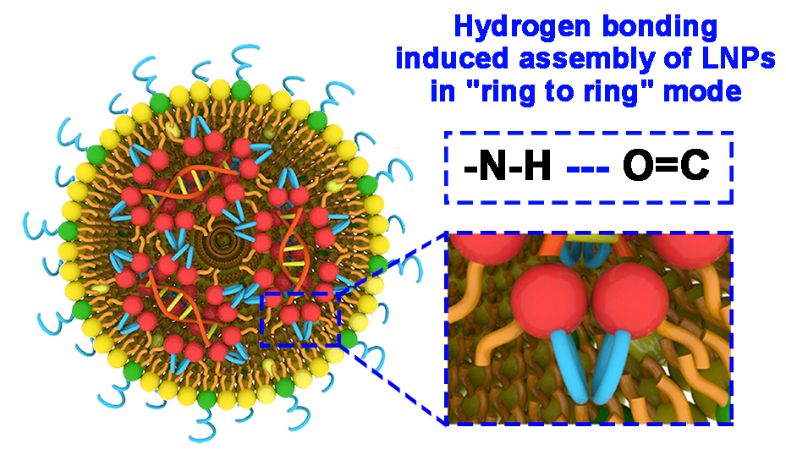
**

**Figure S8.** Schematic illustration of the assembly mechanism of 3D-lipid-based LNPs.

**
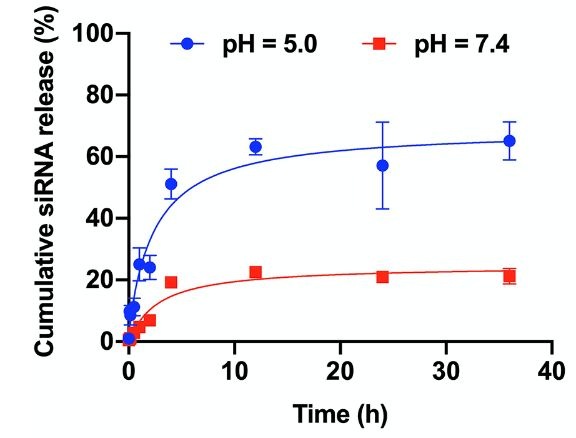
**

**Figure S9.** The siRNA release profile of 2D-LNP/siRNA NPs after mixing with anionic endosomal membranes at pH = 7.4 and 5.0 (n = 3). Data are represented as mean ± SD.

**
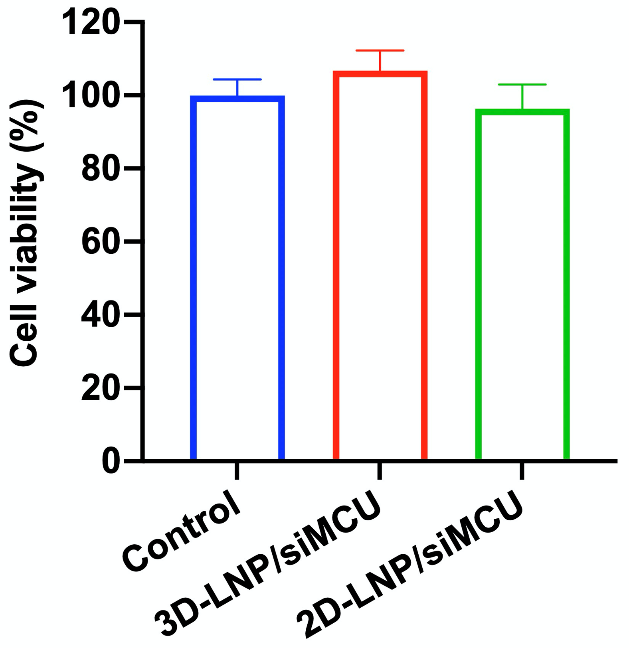
**

**Figure S10.** The cell viability of A549 cells treated with 3D-LNP/siMCU NPs and 2D-LNP/siMCU NPs detected using the CCK-8 assay (n = 6). Data represent mean ± S.D.

**
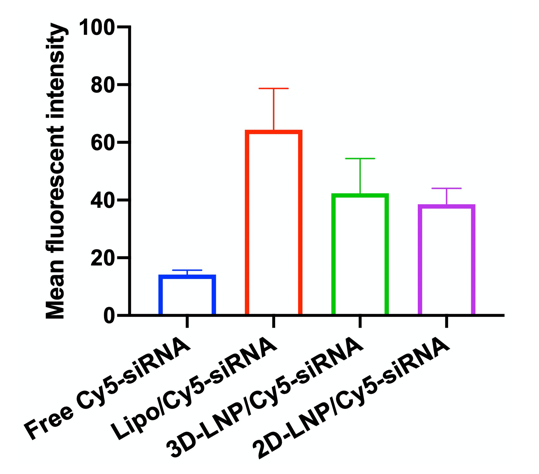
**

**Figure S11.** Cellular uptake and lysosomal escape ability of 3D-LNP/siRNA NPs. Intracellular fluorescence intensities detected using flow cytometry after incubation of BLM-induced A549 cells with Cy5-siRNA-loaded NPs for 6 h (n = 3).

**
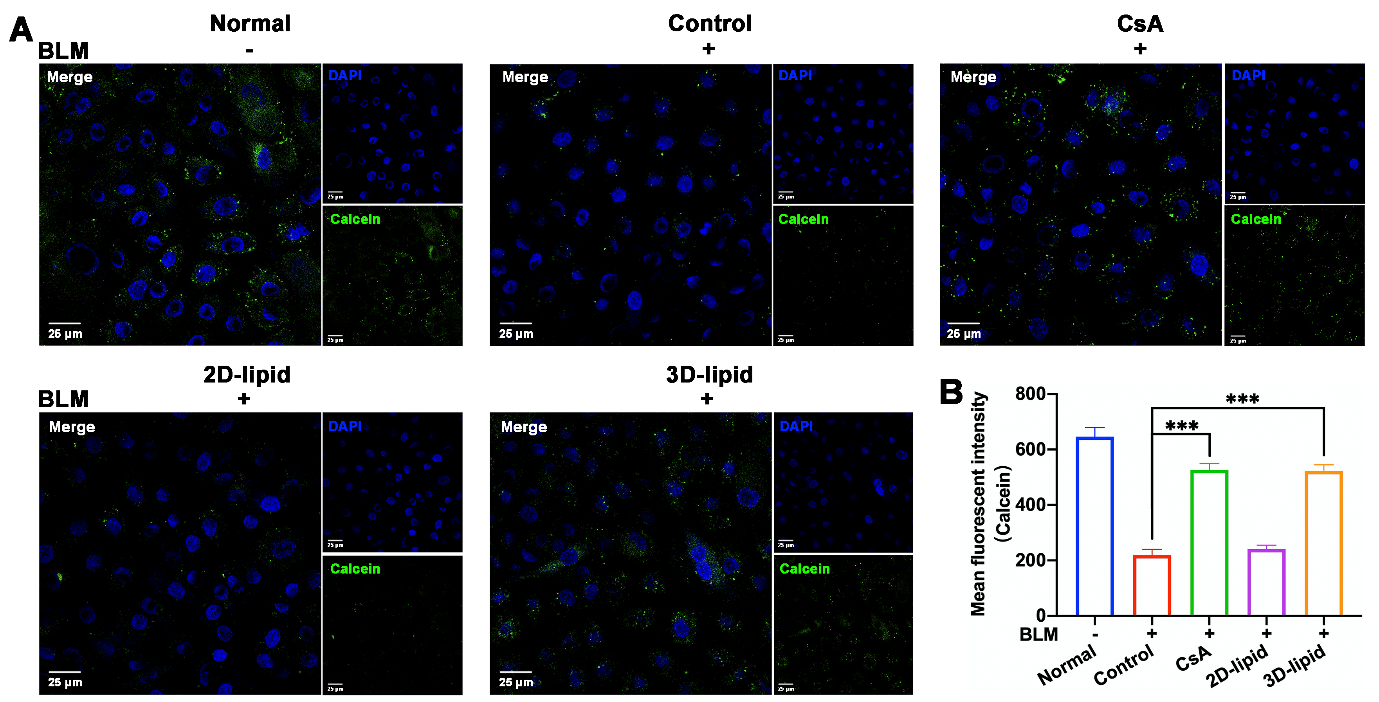
**

**Figure S12.** In Vitro inhibitory effects of 3D-lipid on mPTP opening. The mPTP opening level in BLM-A549 cells detected using CLSM imaging (A) and flow cytometry (B) by the CoCl_2_-calcein-AM fluorescence-quenching method (n = 3). The nucleus was stained with DAPI (blue). Scale bar, 25 μm. Data represent mean ± S.D. **P* < 0.05, ***P* < 0.01, and ****P* < 0.001.

**
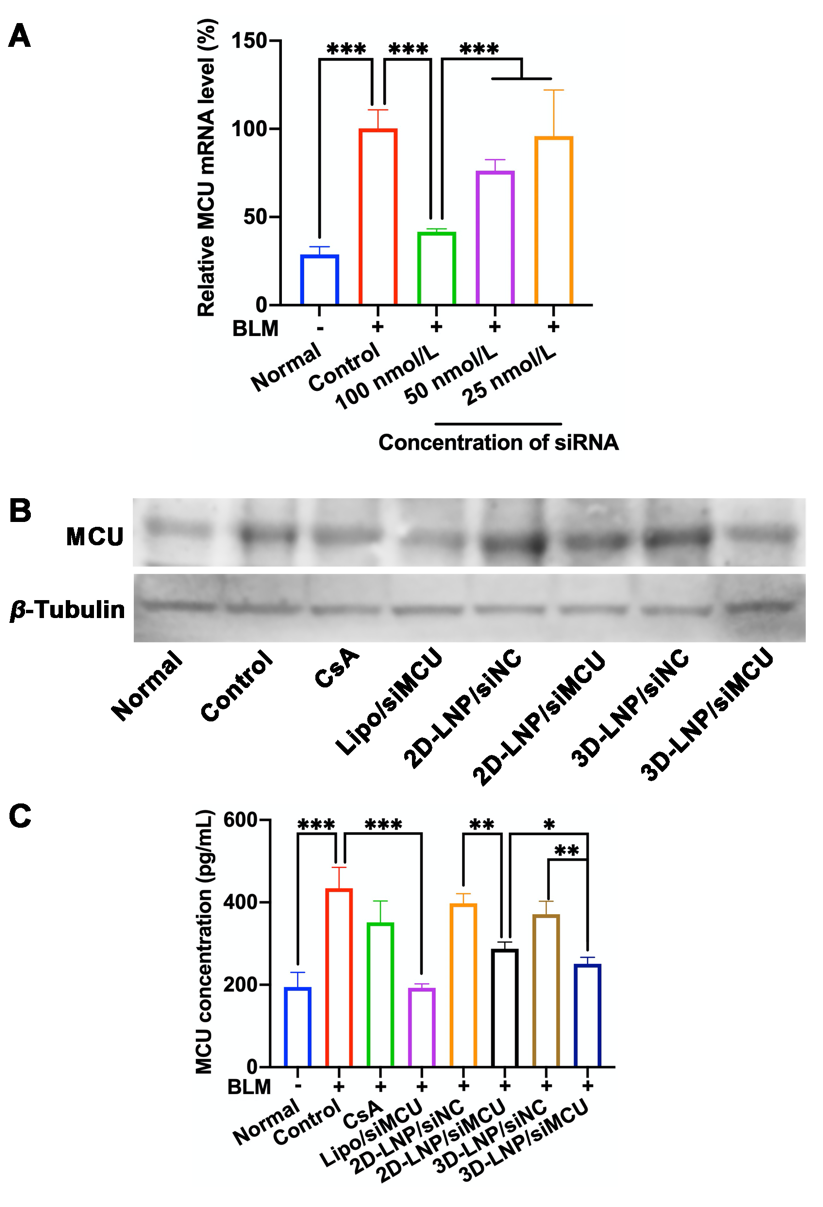
**

**Figure S13.** The MCU expression levels in BLM-induced A549 cells treated with different formulations. (A) The relative MCU mRNA levels in BLM-induced A549 cells after treatment with gradient concentration of siMCU in 3D-LNP/siMCU NPs (n = 3). The MCU expression levels were detected by Western Blot (B) and an ELISA kit (C) (n = 3). Data represent mean ± S.D. **P* < 0.05, ***P* < 0.01, and ****P* < 0.001.

**
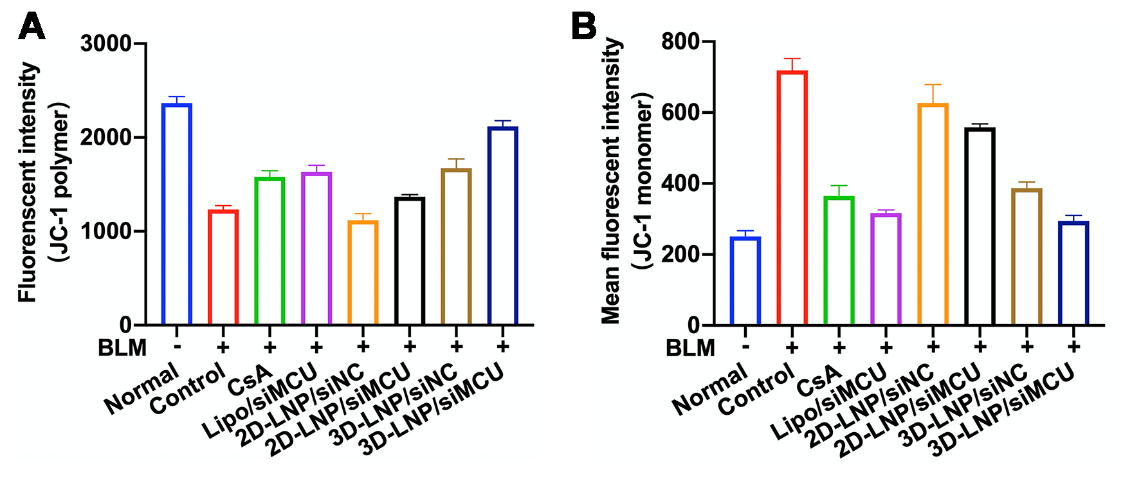
**

**Figure S14.** Flow cytometry was used to evaluate the ΔΨm in BLM-induced A549 cells treated with different formulations. The fluorescent intensities of JC-1 polymer (A) and JC-1 monomer (B) detected by flow cytometry (n = 3). Data represent mean ± S.D.

**
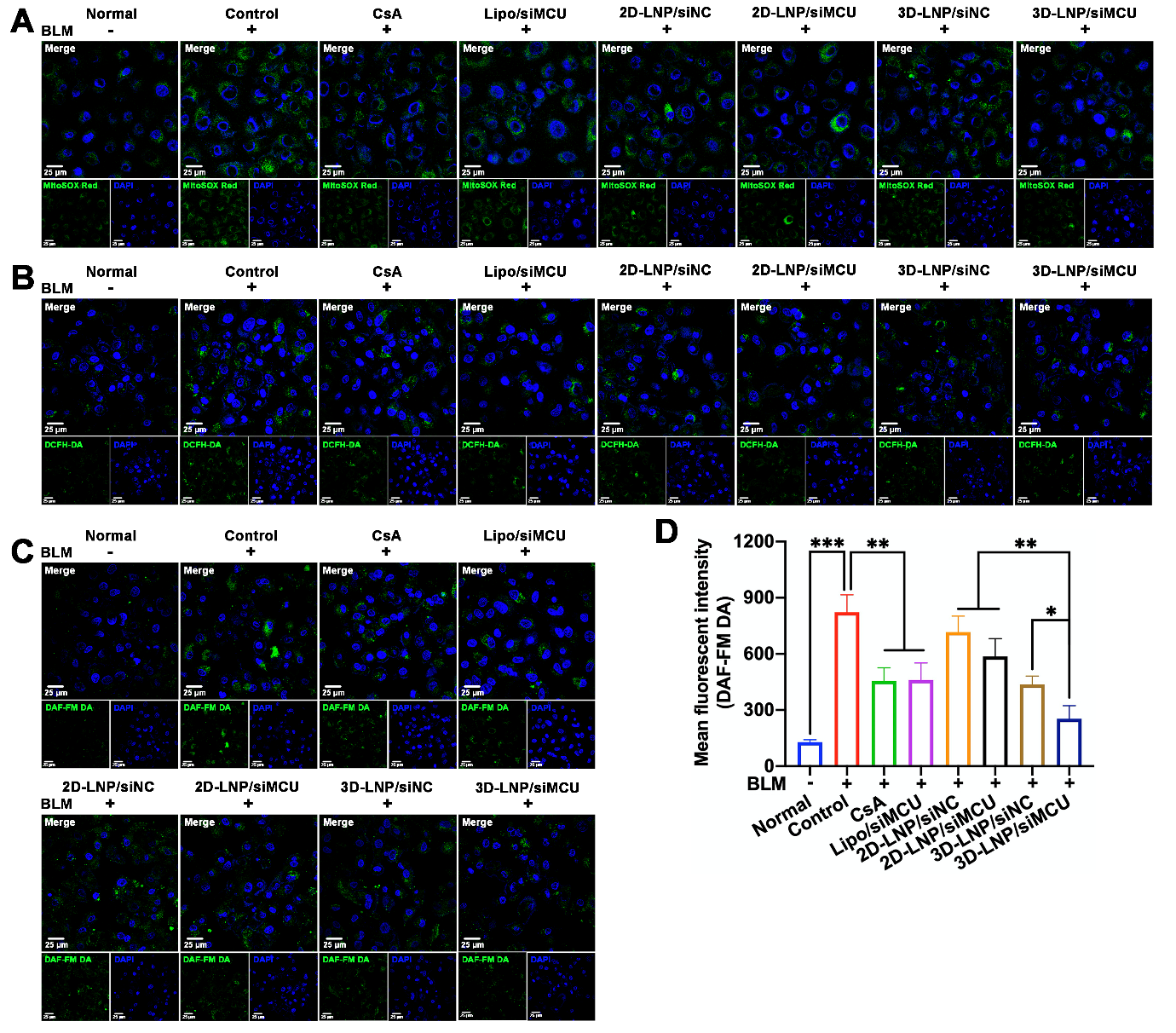
**

**Figure S15.** In vitro evaluation of 3D-LNP/siMCU NPs on BLM-induced A549 cells. CLSM imaging was used to detect the mtROS (A), intracellular ROS (B) and NO (C) levels in BLM-induced A549 cells treated with different formulations. The nucleus was stained with DAPI (blue). Scale bar, 25 μm; (D) Flow cytometry was used to detect the NO level in BLM-induced A549 cells treated with different formulations (n = 3). Data represent mean ± S.D. **P* < 0.05, ***P* < 0.01, and ****P* < 0.001.

**
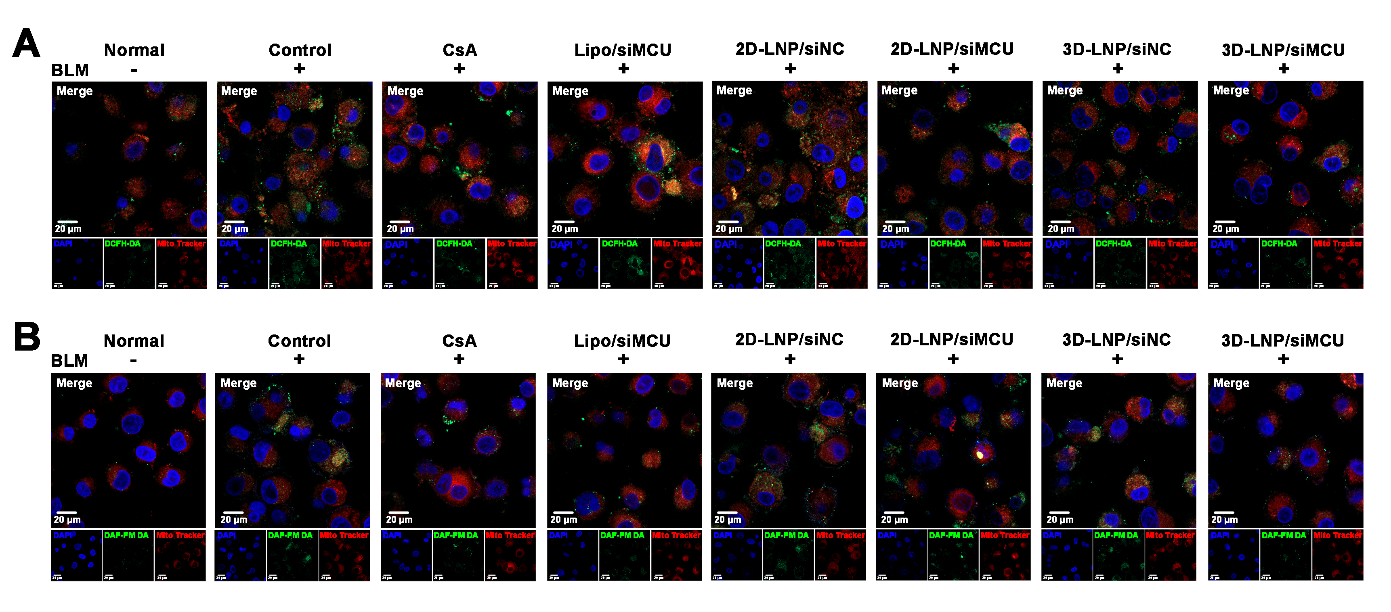
**

**Figure S16.** CLSM imaging was used to evaluate the release of ROS and NO from mitochondrion. CLSM imaging was used to detect the ROS (A) and NO (B) levels in mitochondrion and cytoplasm. The mitochondrion was stained by MitoTracker Red (red). The nucleus was stained by DAPI (blue). Scale bar, 20 μm.

**
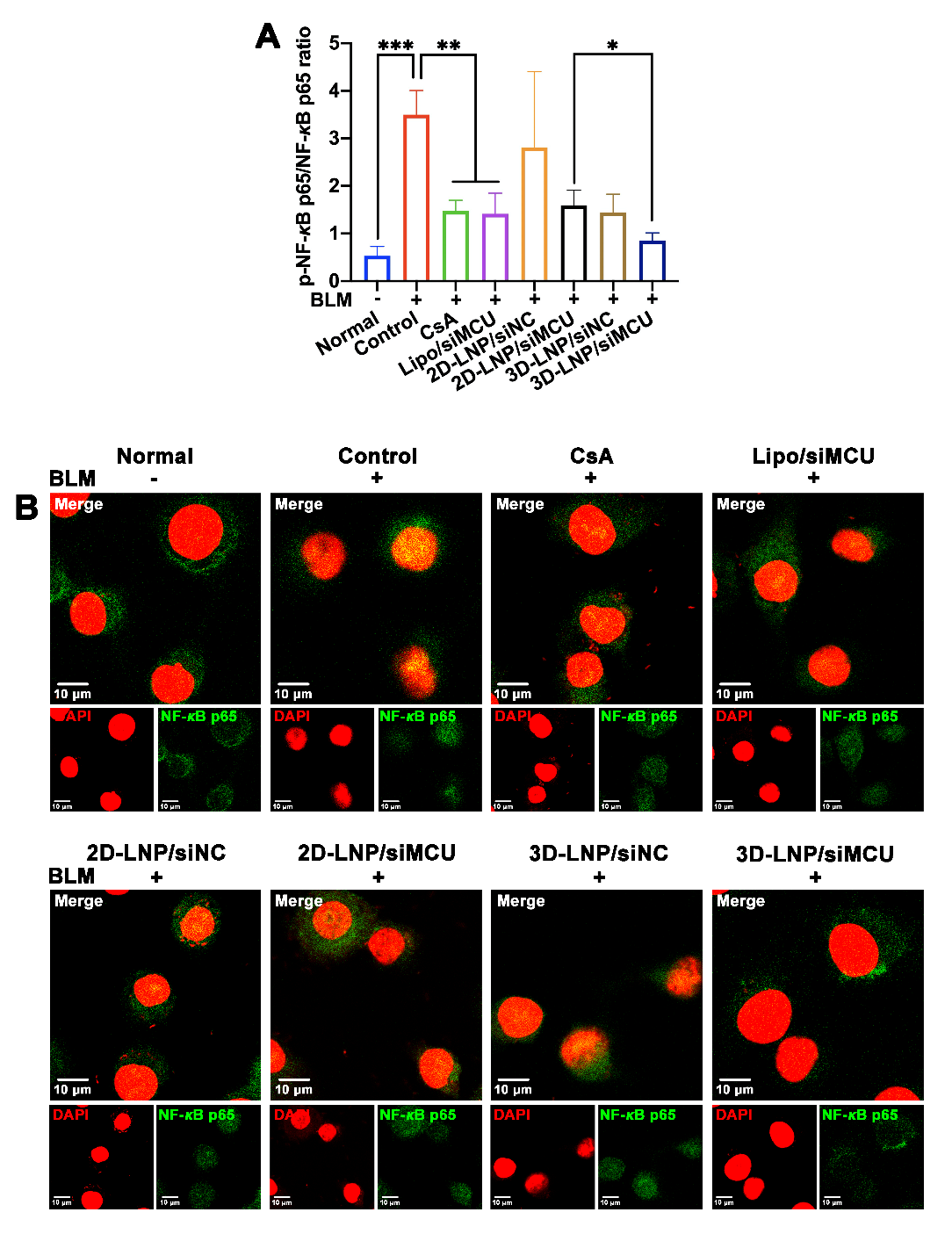
**

**Figure S17.** The effects of 3D-LNP/siMCU NPs on the phosphorylation and translocation of NF-*κ*B p65. (A) ELISA kits were used to detect the p-NF-*κ*B p65/NF-*κ*B p65 ratio in BLM-induced A549 cells treated with different formulations (n = 3); (B) CLSM imaging of nuclear translocation of NF-*κ*B p65. The nucleus was stained with DAPI (red). Scale bar, 10 μm. Data represent mean ± S.D. **P* < 0.05, ***P* < 0.01, and ****P* < 0.001.

**
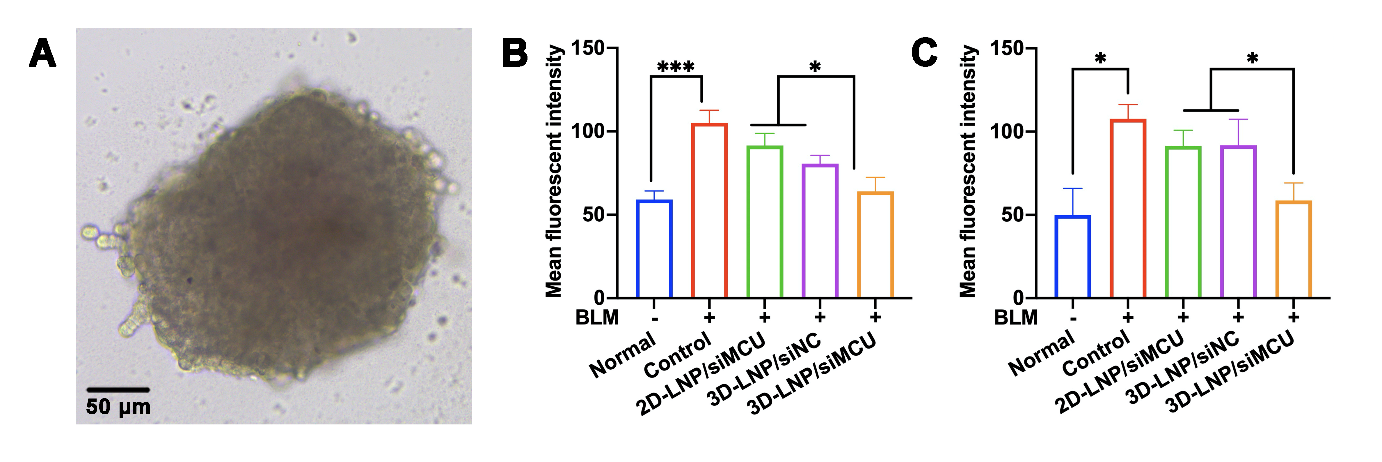
**

**Figure S18.** The effects of 3D-LNP/siMCU NPs on BLM-induced 3D multicellular spheroid models. (A) Optical image of 3D multicellular lung spheroid model. Scale bar, 50 μm; Semi-quantitative analysis of immunofluorescence imaging to detect the expression levels of TGF-*β* (B) and *α*-SMA (C) in 3D multicellular lung spheroid models (n = 3). Data represent mean ± S.D. **P* < 0.05, ***P* < 0.01, and ****P* < 0.001.

**
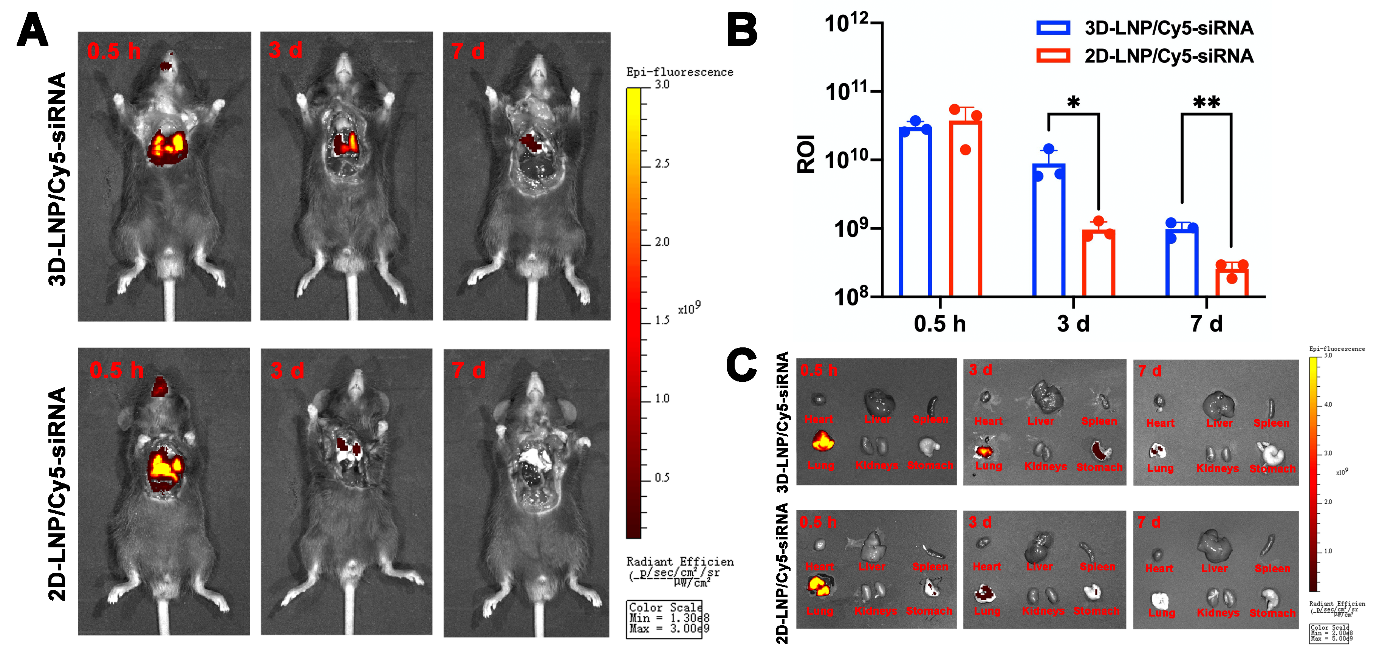
**

**Figure S19.** The biodistribution of NPs. (A) Real-time in vivo imaging of the mice after pulmonary administration of Cy5-siRNA loaded NPs. Radiant Efficiency × 10^9^: (p/sec/cm^2^/sr)/(μW/cm^2^); (B) The ROI in the lung tissues detected using a in vivo imaging system (n = 3); (C) Real-time in vivo imaging of collected organs (heart, liver, spleen, lung, kidneys and stomach). Radiant Efficiency × 10^9^: (p/sec/cm^2^/sr)/(μW/cm^2^). Data represent mean ± S.D. **P* < 0.05, ***P* < 0.01, and ****P* < 0.001.


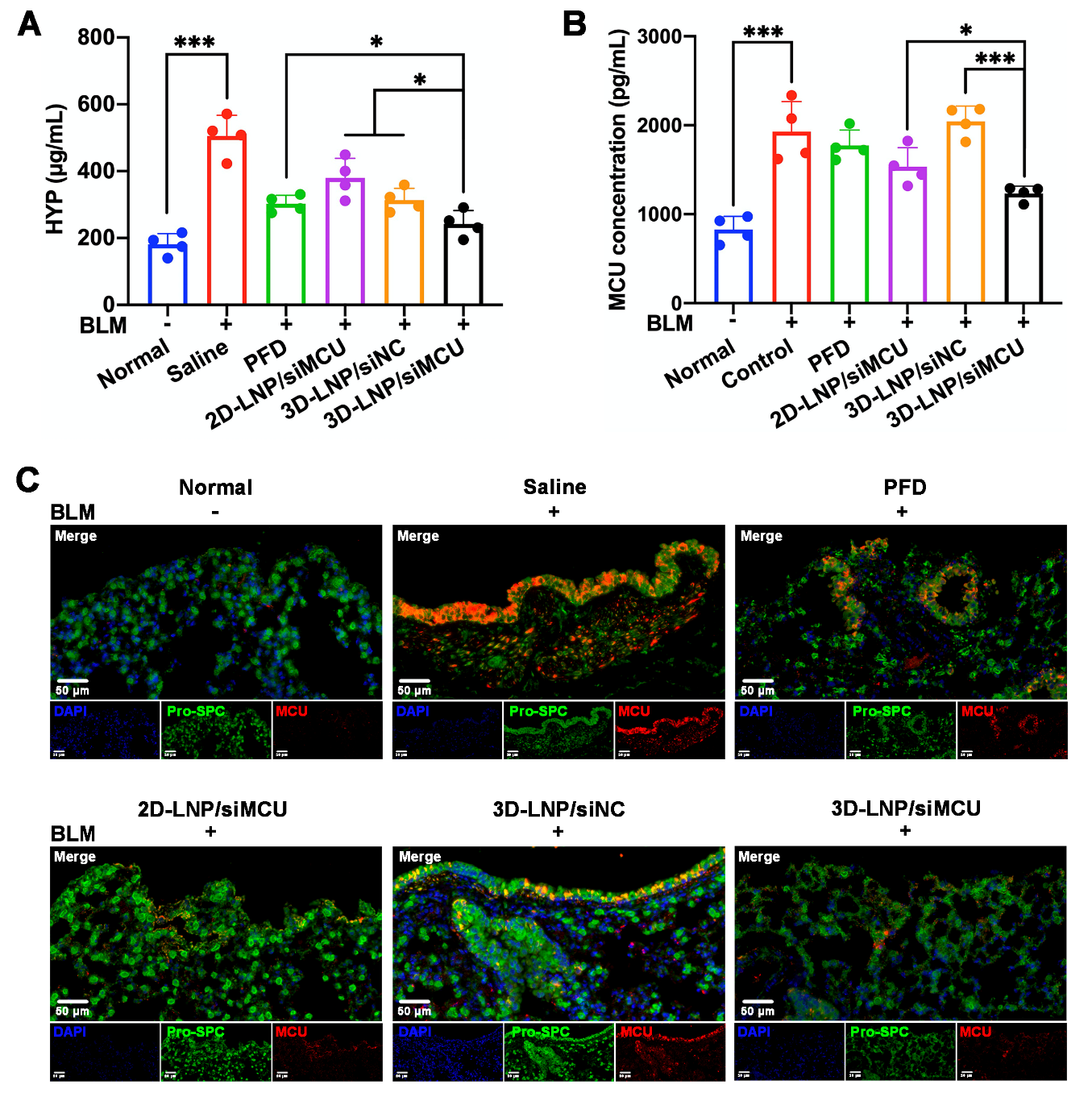


**Figure S20.** The in vivo therapeutic effect of 3D-LNP/siMCU NPs on BLM-induced PF mouse models. (A) The HYP content in the lung tissues isolated from mice detected using a HYP detection kit (n = 4); (B) The MCU expression level in the lung tissues detected using an ELISA kit (n = 4); (C) Representative immunofluorescence images of lung tissues isolated from mice to analyze the MCU (red) expression in ACEIIs (using pro-SPC as a marker, green). The nucleus was stained with DAPI (blue). Scale bar, 50 μm. Data represent mean ± S.D. **P* < 0.05, ***P* < 0.01, and ****P* < 0.001.


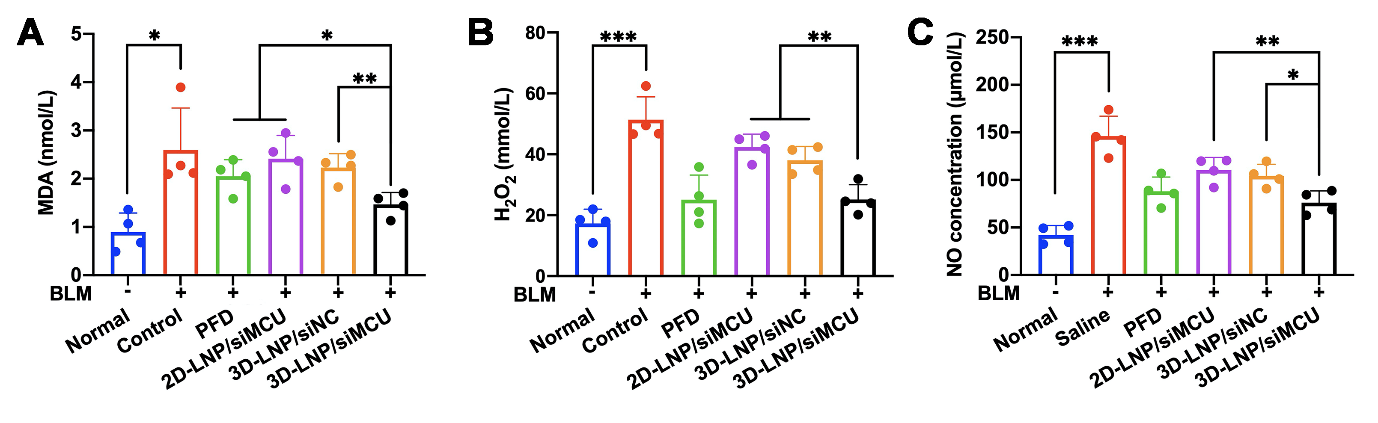


**Figure S21.** The in vivo therapeutic effect of 3D-LNP/siMCU NPs on BLM-induced PF mouse models. (A) The MDA level in lung tissues isolated from mice detected using an MDA detection kit (n = 4); (B) The H_2_O_2_ level in lung tissues isolated from mice detected by H_2_O_2_ a detection kit (n = 4); (C) The NO level in lung tissues isolated from mice detected by Griess assay (n = 4). Data represent mean ± S.D. **P* < 0.05, ***P* < 0.01, and ****P* < 0.001.

**
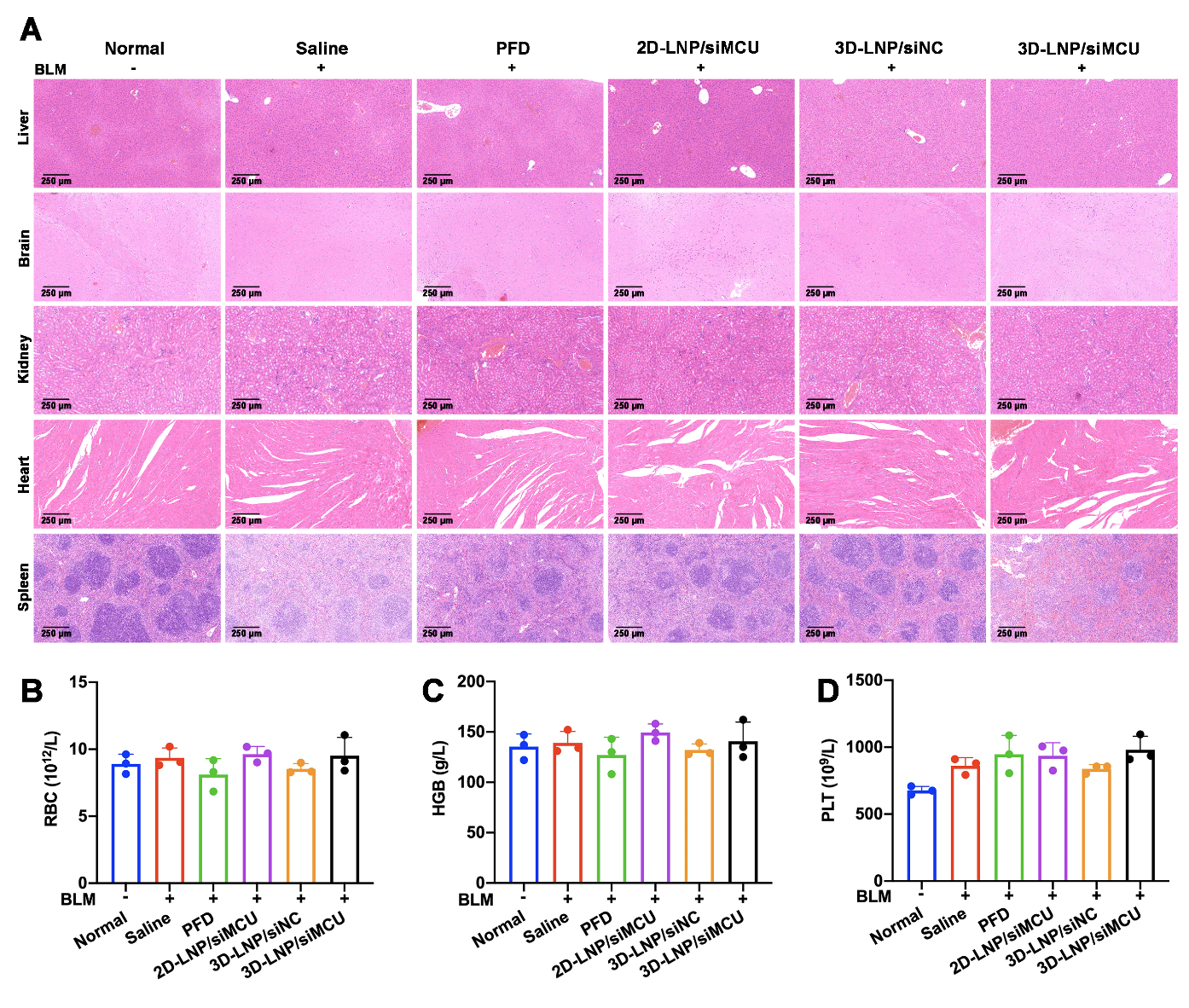
**

**Figure S22.** The in vivo safety of 3D-LNP/siMCU NPs on BLM-induced PF mouse models. (A) H&E staining images of major organs from the mice treated with different formulations. Scale bar, 250 μm; The RBC (B), HGB (C) and PLT (D) levels detected using blood routine test (n = 3). Data represent mean ± S.D.

**
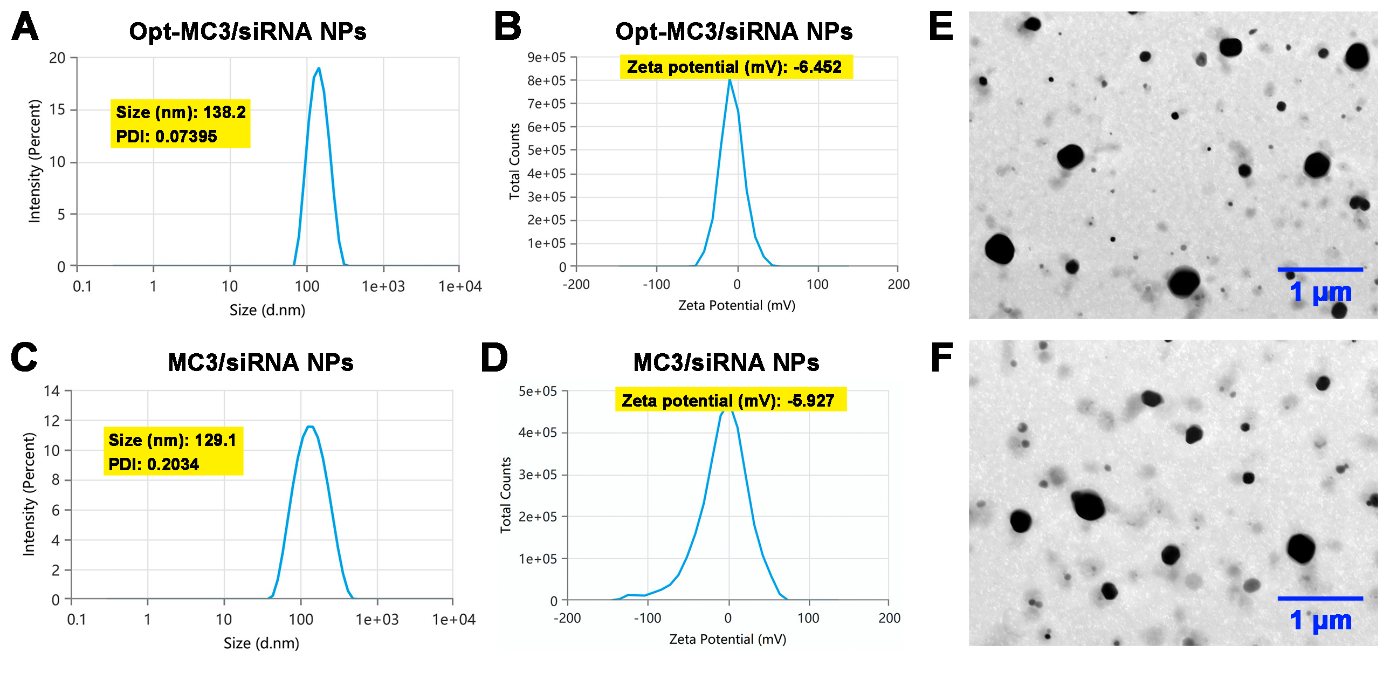
**

**Figure S23.** The comparison of stability between Opt-3D-LNP/siRNA NPs and MC3/siRNA NPs. The particle size (A) and zeta potential (B) of Opt-MC3/siRNA NPs; The particle size (C) and zeta potential (D) of MC3/siRNA NPs; Representative STEM images of Opt-MC3/siRNA NPs (E) and MC3/siRNA NPs (F). Scale bar, 1 μm.


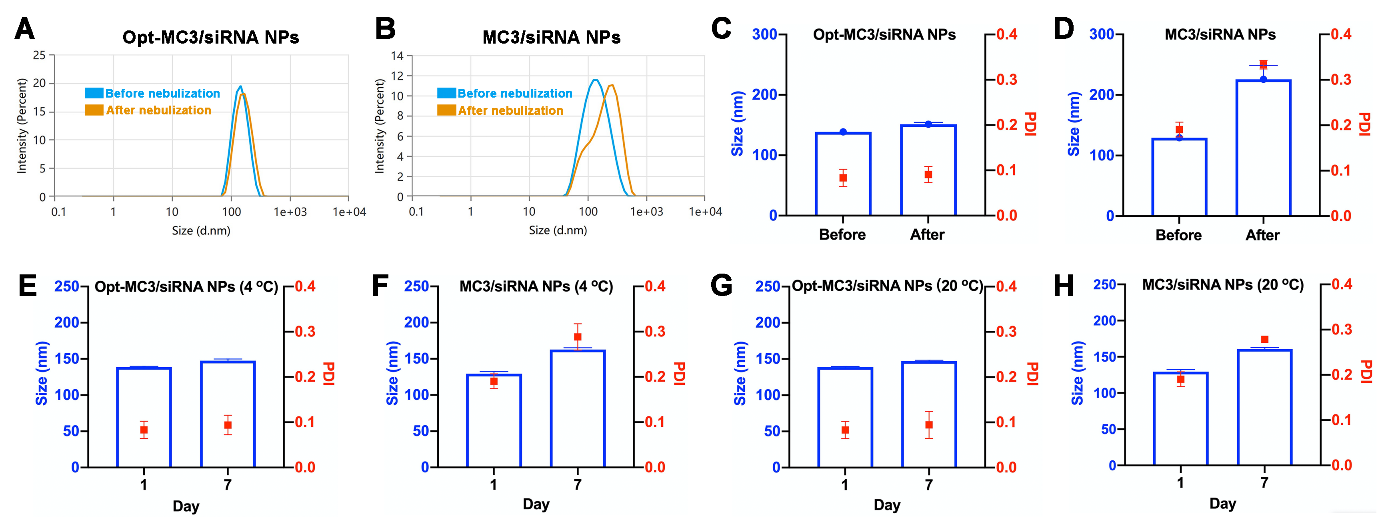


**Figure S24.** The comparison of stability between Opt-3D-LNP/siRNA NPs and MC3/siRNA NPs. The size distribution of Opt-MC3/siRNA NPs (A) and MC3/siRNA NPs (B) before and after nebulization; The particle size and PDI of Opt-MC3/siRNA NPs (C) and MC3/siRNA NPs (D) before and after nebulization (n = 3); The particle size and PDI of Opt-MC3/siRNA NPs (E) and MC3/siRNA NPs (F) after 7-day storage at 4 ^o^C (n = 3); The particle size and PDI of Opt-MC3/siRNA NPs (G) and MC3/siRNA NPs (H) after 7-day storage at 20 ^o^C (n = 3). Data represent mean ± S.D.


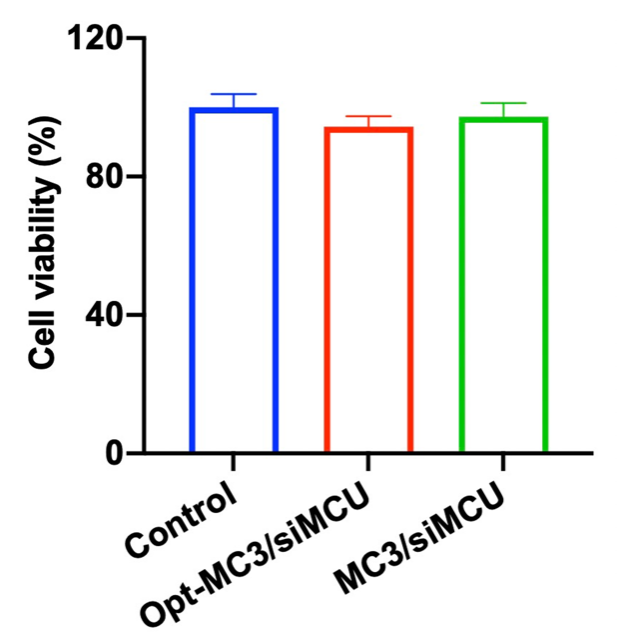


**Figure S25.** The cell viability of A549 cells treated with Opt-MC3/siMCU NPs and MC3/siMCU NPs detected using the CCK-8 assay (n = 6). Data represent mean ± S.D.


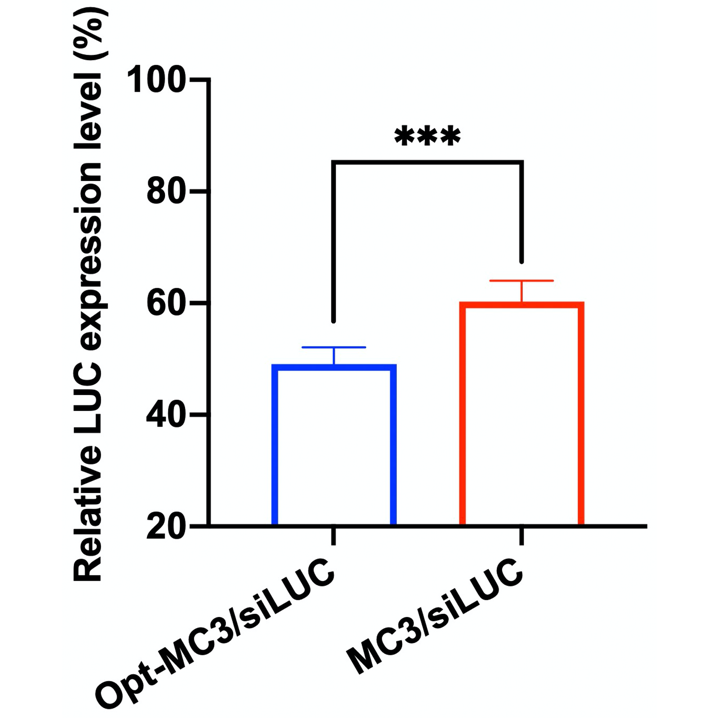


**Figure S26.** The relative LUC expression levels in A549-LUC cells treated with Opt-MC3/siLUC NPs and MC3/siLUC NPs (n = 6). Data represent mean ± S.D. **P* < 0.05, ***P* < 0.01, and ****P* < 0.001.

**
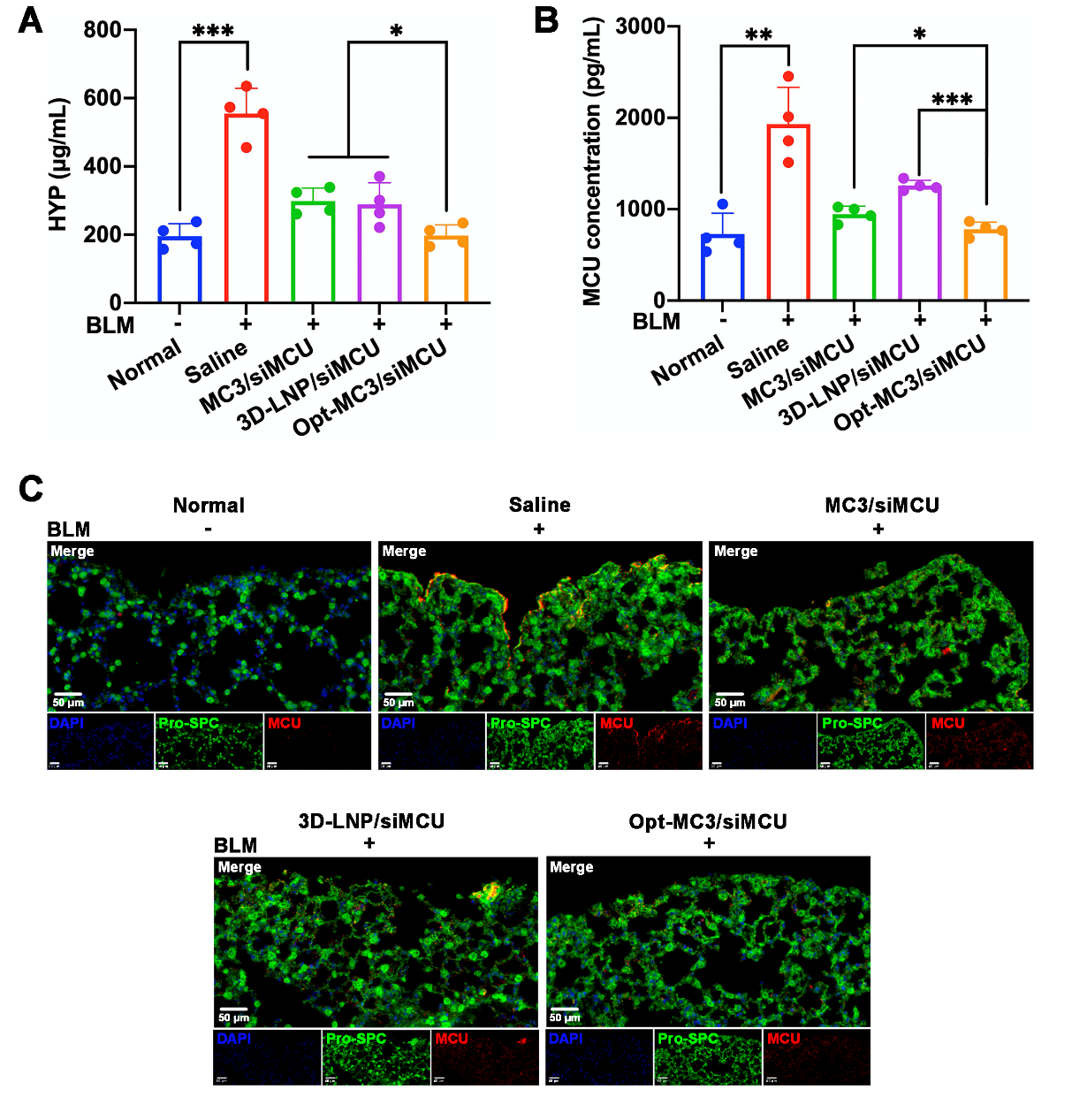
**

**Figure S27.** The in vivo therapeutic effect of Opt-MC3/siMCU NPs on BLM-induced PF mouse models. (A) The HYP content in lung tissues isolated from mice detected using a HYP detection kit (n = 4); (B) The MCU expression level in lung tissues detected using an ELISA kit (n = 4); (C) Representative immunofluorescence images of lung tissues isolated from mice to analyze the MCU (red) expression in ACEIIs (using pro-SPC as a marker, green). The nucleus was stained with DAPI (blue). Scale bar, 50 μm. Data represent mean ± S.D. **P* < 0.05, ***P* < 0.01, and ****P* < 0.001.

**
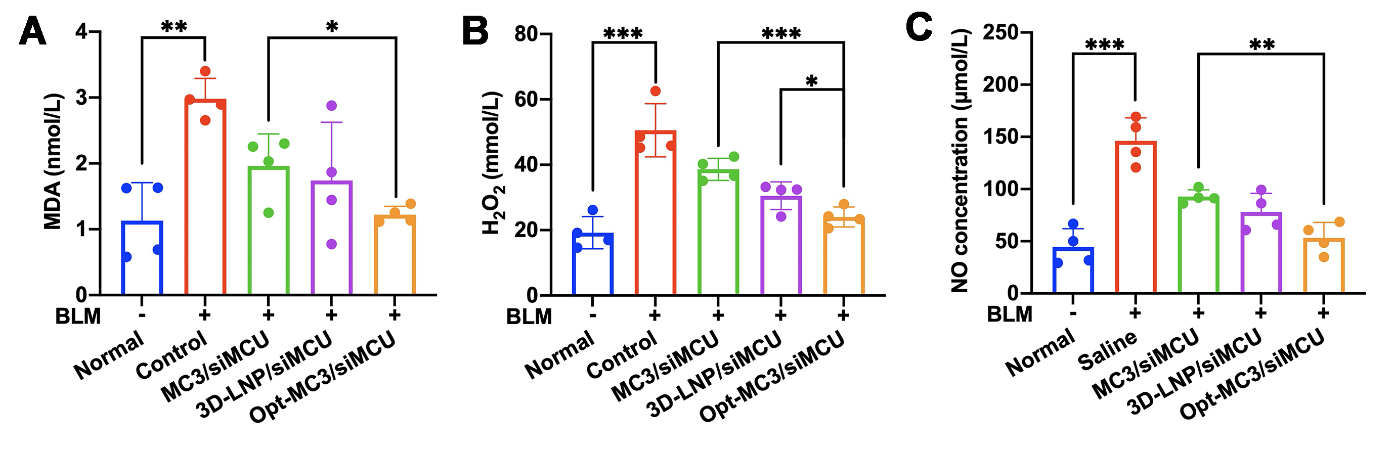
**

**Figure S28.** The in vivo therapeutic effect of 3D-LNP/siMCU NPs on BLM-induced PF mouse models. (A) The MDA level in lung tissues isolated from mice detected using an MDA detection kit (n = 4); (B) The H_2_O_2_ level in lung tissues isolated from mice detected by a H_2_O_2_ detection kit (n = 4); (C) The NO level in lung tissues isolated from mice detected by Griess assay (n = 4). Data represent mean ± S.D. **P* < 0.05, ***P* < 0.01, and ****P* < 0.001.

**
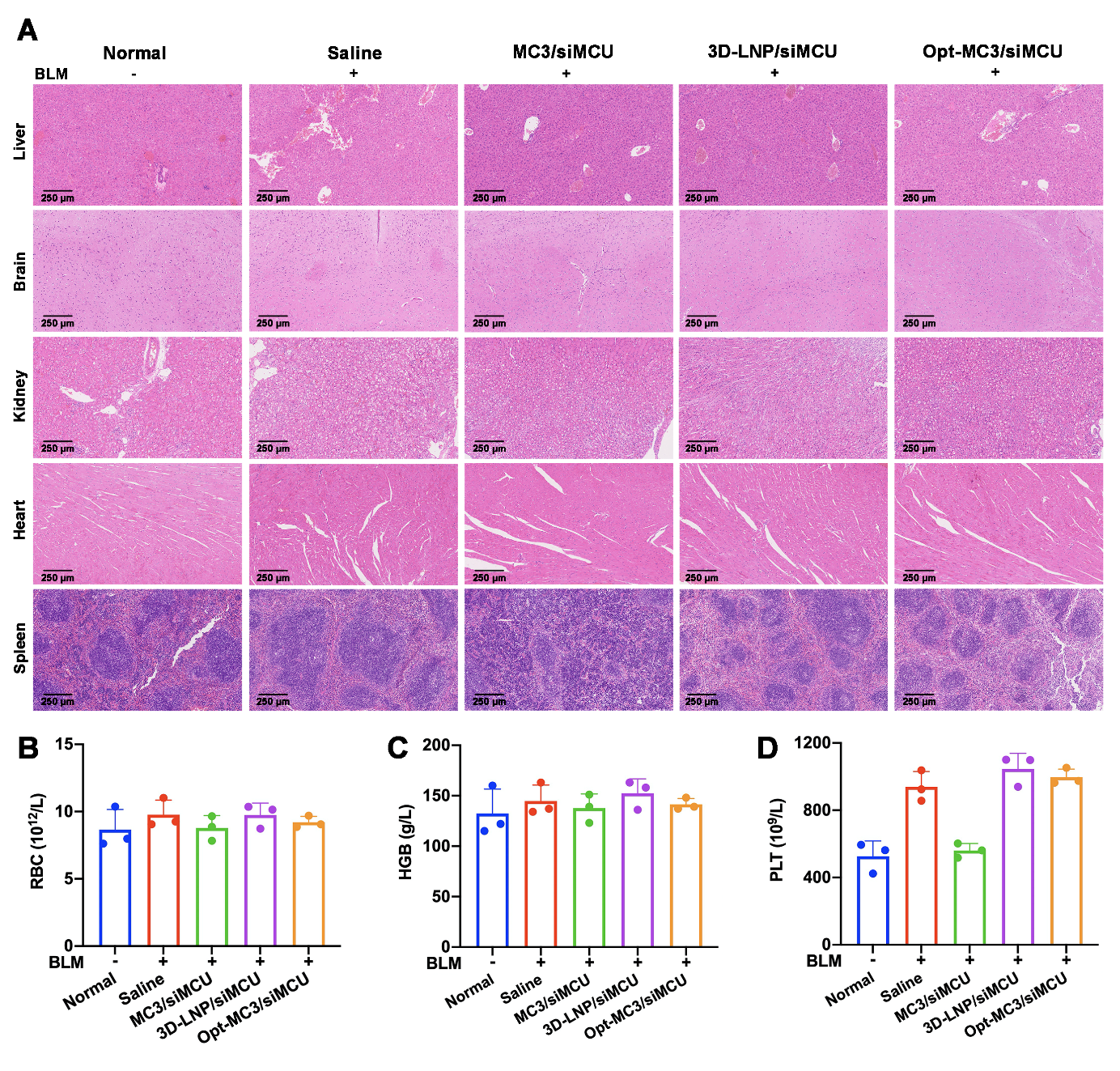
**

**Figure S29.** The in vivo safety of Opt-MC3/siMCU NPs on BLM-induced PF mouse models. (A) H&E staining images of major organs from the mice treated with different formulations. Scale bar, 250 μm; The RBC (B), HGB (C) and PLT (D) levels detected using blood routine test (n = 3). Data represent mean ± S.D.

**Table S1. The formulations of 3D-LNP/siRNA NPs and 2D-LNP/siRNA NPs**

|  | **Molar% (molar ratios)** | | | | | **siRNA encapsulation efficiency (%)** |
| --- | --- | --- | --- | --- | --- | --- |
|  | **3D-lipid** | **2D-lipid** | **DSPC** | **Cholesterol** | **DMG-PEG2000** |  |
| **3D-LNP/siRNA NPs** | **30** |  | **35** | **33.5** | **1.5** | **73.48 ± 0.03** |
| **2D-LNP/siRNA NPs** |  | **30** | **35** | **33.5** | **1.5** | **63.37 ± 0.07** |

**Table S2. The formulations of MC3/siRNA NPs and Opt-MC3/siRNA NPs**

|  | **Molar% (molar ratios)** | | | | | **siRNA encapsulation efficiency (%)** |
| --- | --- | --- | --- | --- | --- | --- |
|  | **MC3** | **DSPC** | **Cholesterol** | **DMG-PEG2000** | **3D-lipid** |  |
| **MC3/siRNA NPs** | **50** | **15** | **33.5** | **1.5** |  | **89.51 ± 0.02** |
| **Opt-MC3/siRNA NPs** | **50** | **15** | **33.5** | **1.5** | **50** | **90.75 ± 0.00** |

**siRNA sequence**

| **siNC** | **Sense strand** | **5'-UUCUCCGAACGUGUCACGUTT-3'** |
| --- | --- | --- |
|  | **Antisense strand** | **5'-ACGUGACACGUUCGGAGAATT-3'** |
| **siLUC (human)** | **Sense strand** | **5'-GAUUAUGUCCGGUUAUGUATT-3'** |
|  | **Antisense strand** | **5'-UACAUAACCGGACAUAAUCTT-3'** |
| **siMCU (mouse)** | **Sense strand** | **5'-CGACCUAGAGAAAUACAAUTT-3'** |
|  | **Antisense strand** | **5'-AUUGUAUUUCUCUAGGUCGTT-3'** |
| **siMCU (human)** | **Sense strand** | **5'-GUACGAAUUGAGAUUAGCATT-3'** |
|  | **Antisense strand** | **5'-UGCUAAUCUCAAUUCGUACTT-3'** |

**Primer sequence**

| ***β*-Actin (mouse)** | **Forward** | **5'-GGTCATCACTATTGGCAACG-3'** |
| --- | --- | --- |
|  | **Reverse** | **5'-ACGGATGTCAACGTCACACT-3'** |
| **MCU (mouse)** | **Forward** | **5'-AAAGGAGCCAAAAAGTCACG-3'** |
|  | **Reverse** | **5'-AACGGCGTGAGTTACAAACA-3'** |
| **SLUG (mouse)** | **Forward** | **5'-TCTGCAGACCCACTCTGATG-3'** |
|  | **Reverse** | **5'-AGCAGCCAGACTCCTCATGT-3'** |
| **SNAI1 (mouse)** | **Forward** | **5'-CACACGCTGCCTTGTGTCT-3'** |
|  | **Reverse** | **5'-GGTCAGCAAAAGCACGGTT-3'** |
| **FN1 (mouse)** | **Forward** | **5'-GAGCTATCCATTTCACCTTCAGA-3'** |
|  | **Reverse** | **5'-TTGTTCGTAGACACTGGAGAC-3'** |
| **GAPDH (human)** | **Forward** | **5'-CTCCCCACACATGCACTTA-3'** |
|  | **Reverse** | **5'-CCTAGTCCCAGGGCTTTGATT-3'** |
| **MCU (human)** | **Forward** | **5'-TCCAGAAGCCAGAGACAGAC-3'** |
|  | **Reverse** | **5'-TGTCGGAGAGGCAGATGTAC-3'** |
| **SLUG (human)** | **Forward** | **5'-ACGCCCAGCTACCCAATG-3'** |
|  | **Reverse** | **5'-CGCCCCAAAGATGAGGAGTA-3'** |
| **SNAI1 (human)** | **Forward** | **5'-CCCCAATCGGAAGCCTAACT-3'** |
|  | **Reverse** | **5'-GCTGGAAGGTAAACTCTGGATTAGA-3'** |
| **FN1 (human)** | **Forward** | **5'-CCATCGCAAACCGCTGCCAT-3'** |
|  | **Reverse** | **5'-AACACTTCTCAGCTATGGGCTT-3'** |
| **MT-CO1 (human)** | **Forward** | **5'-GCAACCTCAACACCACCTTC-3'** |
|  | **Reverse** | **5'-ATTCCGAAGCCTGGTAGGAT-3'** |
| **MT-CO2 (human)** | **Forward** | **5'-TTCCTAGTCCTGTATGCCCT-3'** |
|  | **Reverse** | **5'-TATACCCCCGGTCGTGTAGC-3'** |
| **TOMM5 (human)** | **Forward** | **5'-TGGACCCGGAGGAGATGAAA-3'** |
|  | **Reverse** | **5'-GGAGGGCCACGTAGATGAGA-3'** |
| **TOMM6 (human)** | **Forward** | **5'-CTGCTGGCTCGGCTAATGAAA-3'** |
|  | **Reverse** | **5'-ATCAGTGGCAAAGCGGTAGAC-3'** |
